# New Tirucallane-Type Triterpenes Isolated from *Commiphora oddurensis* from Ethiopia with Anticancer Activity

**DOI:** 10.1021/acsomega.5c03203

**Published:** 2025-07-30

**Authors:** Taame G. Seyoum, Zhenming Du, Siming Wang, Wen Lu, Qiyue Mao, Xiaoxiao Yang, Aman Dekebo, Ermias Dagne, Paulos Yohannes, Binghe Wang

**Affiliations:** † Department of Chemistry, 37602 Addis Ababa University, Miazia 27 Sq., P.O. Box 30270 Addis Ababa, Ethiopia; ‡ Department of Chemistry, 1373 Georgia State University, 50 Decatur St, Atlanta, Georgia 30303, United States; § Perimeter College, Georgia State University, 2101 Womack Road, Dunwoody, Georgia 30338, United States

## Abstract

Three new tirucallane-type triterpenes, tirucalla-7,24-diene-1β,3β-diol (oddurensinoid **B**), tirucalla-7-ene-1β,3β,25-triol (oddurensinoid **H**), and tirucalla-7, 24-diene-3β-ol-1-*O*-β-d-glucopyranoside (oddurensinoid **K**), were isolated from the resin of *Commiphora oddurensis* harvested from Ethiopia, Africa. Their structures were elucidated by one-dimensional (1D) NMR, two-dimensional (2D) NMR, and high-resolution mass spectrometry (HRMS). All three compounds were tested for their anticancer activity against HeLa cell lines. They all exhibit anticancer activity, with oddurensinoid **H** the most potent with IC_50_ of 0.017 mg/mL (36.9 μM).

## Graphical abstract

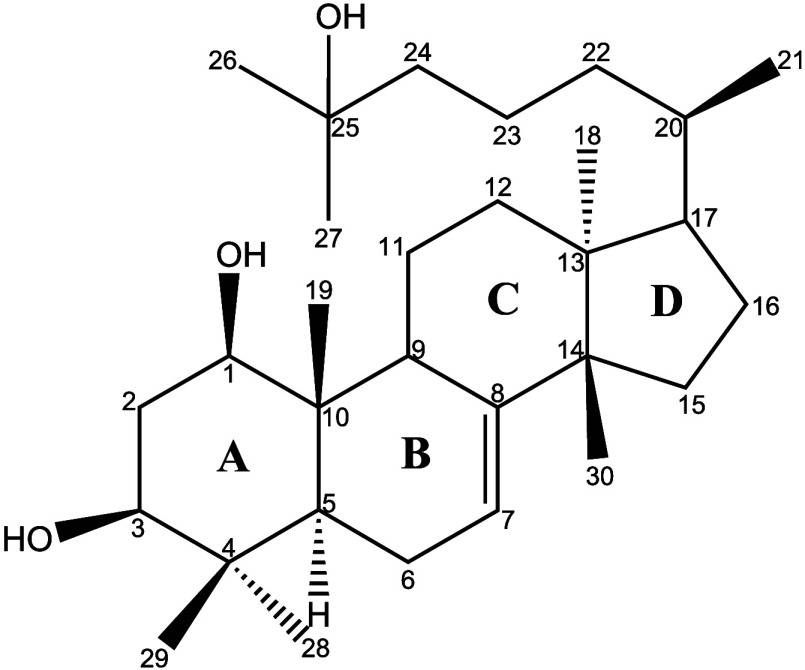

## 1 Introduction

Plants produce resinous exudate that serves as an essential resource in traditional medicines.^1,2^ One notable genus known for such exudates is *Commiphora*, belonging to the family Burseraceae, which comprises over 150 species of trees and shrubs. This genus is primarily distributed across East Africa, the Arabian Peninsula, and India.^3,4^ Of the more than 50 *Commiphora* species found in Ethiopia, approximately 25% are endemic.^4^ The exudates from *Commiphora* species contain volatile oils, alcohol-soluble resins, water-soluble gums, and gum resins that are partially soluble in both alcohol and water.^3,5,6^ A well-known example is myrrh, a gum resin obtained from the *Commiphora myrrha* (also referred to as *myrrha* (Nees) Engl.), locally known in Amharic as *kerbe* (myrrh). Myrrh is widely distributed in Ethiopia, Somalia, and Kenya. It is traditionally used as a fragrance incense across various cultures and plays an important role in traditional medicine systems of East Africa, Arabia, India, China, and Europe.^3,7–11^ Myrrh has been used to treat a wide range of ailments, including inflammation, rheumatism, allergies, colds, coughs, asthma, gastrointestinal disorders, diarrhea, headaches, insect bites, wounds, and even to repel snakes.^12–16^ The chemical composition of myrrh has been extensively studied, with monoterpenoid and sesquiterpenoids identified as its predominant bioactive constituents.^17–22^

In addition to *Commiphora myrrha*, the chemical and biological profiles of numerous other species within the *Commiphora* genus, such as *Commiphora opobalsamum*, *Commiphora mukul*, *Commiphora kua*, *Commiphora confusa*, *Commiphora sphaerocarpa*, *Commiphora Africana*, *Commiphora guidottii*, *Commiphora wightii*, *Commiphora incisa*, *Commiphora merkeri*, *Commiphora dalzielii*, *Commiphora abyssinica*, *Commiphora holtziana*, *Commiphora pyracanthoides*, *Commiphora erlangeriana*, and *Commiphora erythraea*, have been extensively studied. The species have demonstrated a wide range of biological activities, including anti-inflammatory, cytotoxic, hepatoprotective, neuroprotective, antimicrobial, antiulcerative colitis, wound healing, and lipid accumulation inhibitory effects.^14,15,22^ To date, more than 300 bioactive compounds have been identified across the genus, including terpenoids, steroids, lignans, flavonoids, carbohydrates, and long-chain aliphatic alcohols, with sesquiterpenes and triterpenes being the most predominant classes.^13,14^ Owing to the genus’s profound cultural, medicinal, and economic importance, phytochemical and pharmacological research of *Commiphora* species continues to grow steadily.

As part of an ongoing search for bioactive secondary natural products from the genus *Commiphora*, we report the isolation and structural elucidation of three new tirucallane-type triterpenes from the resin of the relatively recently identified species, *Commiphora oddurensis*,^23^ collected from the Gode district in the Ethiopia Somali regional state. The structures of those compounds were determined by using a combination of advanced spectroscopic techniques, including NMR (^1^H, ^13^C, DEPT, COSY, NOESY, TOCSY, HSQC, HMBC, HSQC-TOCSY, and 2D ^1^H *J*-resolved), mass spectrometry (MS), and IR spectroscopy. All three triterpenes were subsequently evaluated for their cytotoxic activity against human cervical cancer (HeLa) cells, and the results revealed promising anticancer potential.

## 2 Materials and Methods

### 2.1 General Experimental Procedures

All chemicals and solvents used in this study were of laboratory grade. Sonicor (SC-101TH) from Sonicor Instrument Corporation, Copiague, New York, was used during the extraction of the resin material. BüCHI-RE111, Switzerland, and a Vacuum Pump (MZ 2C NT) connected to a Vacuum Controller (CVC 3000) from VACUUBRAND GMBH + CO KG, Germany, were used to remove solvents under reduced pressure. Silica gel H (5–40 μm, 150 g) without CaSO_4_ from Fluka Chemie AG CH-9470 Buchs, Switzerland, was used for vacuum liquid chromatography (VLC). Wet normal phase column chromatography (CC) was performed using silica gel 60 (70–230 mesh ASTM, 0.063–0.200 mm, Merck). Each sample was dissolved in methanol (MeOH) and adsorbed onto an appropriate amount of silica gel 60 before applying it on top of the preprepared CC. Four column sizes (1.0 cm × 22.0 cm, 3.4 cm × 45.0 cm, 4.2 cm × 20.0 cm, and 5.6 cm × 20.0 cm) were used to isolate and purify compounds. The mobile phase used was a mixture of Petroleum Ether (PE), diethyl ether (CH_3_CH_2_OCH_2_CH_3_, Et_2_O), dichloromethane (CH_2_Cl_2_, DCM), chloroform (CHCl_3_), ethyl acetate (CH_3_O_2_CH_2_CH_3_, EtOAc), and methanol (CH_3_OH, MeOH) in increasing order of polarity. Fractions were collected using vials, and each fraction was monitored using TLC. Preparative TLC (PTLC) plates (20 cm × 20 cm) with 0.5 mm layer thickness were made using a mechanical PTLC maker and silica gel GF254, with 13% calcium sulfate (CaSO_4_) and fluorescent indicator (Fluka Chemie AG CH-9470 Buchs, Switzerland).

### 2.2 Plant Material

The plant material of *Commiphora oddurensis* resin, known by its vernacular name Tubuk in Somali, was collected by Professor Aman Dekebo in October 1997 near the Gode district of the Ogaden region of the Somali regional state, Ethiopia. Voucher specimens were deposited at the National Herbarium of Addis Ababa University, Addis Ababa, Ethiopia, under Voucher Number D61. The botanical identities of the specimen were established by Kaj Vollesen, The Herbarium, Royal Botanic Gardens, Kew, Richmond, Surrey TW9 3AB, U.K., and Prof. Sebsebe Demissew, The National Herbarium, Addis Ababa University, Addis Ababa, Ethiopia. Collecting samples from the wild to investigate their chemical study and evaluate their activity against HeLa cells does not necessitate permission.

### 2.3 Extraction and Isolation

A clean and air-dried resin sample of *Commiphora oddurensis* was mechanically pounded into a powder using a metal mortar and pestle. The powdered resin (100.0 g) was extracted using a mixture of ethyl acetate and methanol (200 mL × 3, 1:1) for 45 min in a hot sonic bath at 40 °C. The heterogeneous mixture was filtered using suction, and the solvent was removed under reduced pressure to yield a yellowish solid crude extract (65.0 g, 65%). From this amount, 60.0 g was applied to VLC and successively partitioned into CHCl_3_ (200 mL × 3), EtOAc (200 mL × 3), and MeOH (200 mL × 3), yielding a yellowish gel (28.0 g, 46.7%), a yellowish-white solid (20.0 g, 34.1%), and a reddish solid (9.0 g, 15.1%) portion, respectively.

Part of the CHCl_3_ portion (20.0 g) was subjected to silica gel CC and was eluted with a gradient of PE/DCM (100:0 → 0:100), DCM/EtOAc (100:0 → 0:100) and EtOAc/MeOH (100:0 → 80:20). Similar eluents were pooled based on the TLC spots to produce five major fractions (Fractions A–E). Recrystallization of the Fraction C (5.8 g) from PE/DCM (4:1 × 3) afforded Myrrhasin (1.1 g). The mother liquor (1.0 g) was placed on top of silica gel CC and separated with a DCM/EtOAc (100:0 → 20:80) solvent mixture to collect five subfractions (Fractions C1–C5). Fr. C3 (0.190 g) was purified using PTLC (95% DCM in MeOH, 60 mL) to furnish oddurensinoid **B** (0.040 g). Furthermore, Fraction E (1.3 g) was subjected to silica gel CC and eluted successively with a gradient of DCM/EtOAc (50:50 → 0:100) and EtOAc/MeOH (100:0 → 80:20) to achieve three subfractions (Fractions E1–E3). Among these, Fraction E3 (0.080g) was allowed to silica gel CC EtOAc/MeOH (100:0 → 80:20) and further purified using PTLC (97% DCM in MeOH), yielding oddurensinoid **H** (0.023 g). On the other hand, a total of five fractions (Fractions A–E) were obtained from the combined EtOAc portion (18.0 g) after application on top of silica gel CC (400 g) and eluted with a solvent mixture of DCM/EtOAc (80:20 → 0:100) and EtOAc/MeOH (100:0 → 80:20) with increasing polarity. Fraction E (1.0 g) was chromatographed on silica gel CC, eluted successively with DCM/EtOAc (100:0 → 20:80) to give five subfractions (Fractions E1–E5). Oddurensinoid K (0.105 g) was obtained from Fraction E2 (0.120 g), which was purified using PTLC (95% DCM in MeOH).

Melting points were measured using the METTLER TOLEDO, FP90 Central Processor as a main display attached to the METTLER TOLEDO, FP82HT Hot-Stage. Ultraviolet (UV) data were generated using PerkinElmer, Lambda 950, UV/vis/NIR spectrometer. Fourier transform infrared (FT-IR) spectra were recorded in potassium bromide (KBr) pellets using Spectrum 65 FT-IR, PerkinElmer in the range 4000–400 cm^–1^ (resolution: 4 cm^–1^, number of scans: 4).

### 2.4 Mass Spectrometry

Electrospray ionization mass spectrometry (ESI-MS) analyses were performed on a Waters Xevo G2-XS mass spectrometer (Waters Corporation, Milford, MA) equipped with an electrospray ionization source in positive-ion mode. The original samples were dissolved in methanol. Each sample (5 μL) was introduced into the ion source through an autosampler with a flow rate of 100 μL/min. The instrument operation parameters were optimized: capillary voltage of 1000 V, sample cone voltage of 20 V, desolvation temperature of 350 °C, and source temperature of 120 °C. Nitrogen was used as the cone and desolvation gas at 25 and 800 L/h pressures, respectively. The spectra were acquired through a complete scan analysis. MassLynx 4.2 software was used for the data acquisition and processing.

### 2.5 NMR Data

Deuterated CHCl_3_ and MeOH were purchased from Sigma-Aldrich. The NMR spectra, including ^1^H, ^13^C, *J*-resolved, DEPT, HSQC, HMBC, NOESY, TOCSY, COSY, and HSQC-TOCSY, were recorded on a Bruker (Avance III HD spectrometer at 25 °C; Bruker Biospin, Billerica, MA). The spectrometer has an operating frequency of 599.84 MHz for ^1^H, and 150.83 MHz for ^13^C. MestReNova, ACD/LABs, and Bruker Topspin were used for spectrum processing. ^1^H and ^13^C chemical shifts (δ) were observed and are reported in parts per million (ppm) relative to the TMS at 0.00 ppm as an internal reference. Some solvents do not contain internal TMS. In those cases, MeOD peaks are used for calibration with methyl group protons calibrated as 3.31 ppm and methyl carbon calibrated as 49.00 ppm. Or CDCl3 is used for proton chemical shift calibration of 7.26 ppm and carbon chemical shift calibration of 77.16 ppm.

### 2.6 Biological and Cytotoxic Activities

The human cervical cancer cells (HeLa cells) were obtained from American Type Culture Collection (ATCC) and cultured in Dulbecco’s modified Eagle’s medium (DMEM, Corning) supplemented with 10% fetal bovine serum (Corning) and 100 unit/mL penicillin, and 0.1 μg/mL streptomycin at 37 °C in a humidified atmosphere in the presence of 5% CO_2_. Cell viability test (cytotoxicity assay) on HeLa was assessed following the protocol using Cell Counting Kit-8 (CCK-8, Dojindo, Japan). Briefly, HeLa cells (ca. 105 cells) in DMEM (100 μL) were seeded into each well of 96-well plates and incubated at 37 °C in a humidified CO_2_ atmosphere (5%) for 24 h. Compounds were dissolved in dimethyl sulfoxide (DMSO, 100 μL), then diluted with the culture medium to various concentrations (final DMSO concentrations 0.5%). The cell incubation medium was replaced with fresh DMEM (100 μL) containing compounds. After 24 h incubation, the medium was removed and washed with phosphate-buffered saline (PBS), then fresh DMEM containing 10 μL of CCK-8 solution was added to each well, and the plate was incubated for an additional 2–4 h at 37 °C before measuring the optical density at 450 nm with a microplate reader (PerkinElmer Victor 2). The cell viability of each well was calculated as a percentage of the untreated control according to the manufacturer’s manual. All tests were performed in triplicate, and IC_50_ values of active compounds were determined by nonlinear regression using GraphPad Prism 9.

## 3 Results and Discussion

From the resin of the newly identified species *Commiphora oddurensis*, over ten triterpenoid compounds were isolated, including the previously reported triterpenoid myrrhasin (Figure 1I).^24^ Building on prior spectroscopic study of myrrhasin^24^ and the euphane-type triperpenoids,^25^ we successfully elucidated the structures of three new compounds: oddurensinoid **B**, oddurensinoid **H**, and oddurensinoid **K** (Figure 1II–IV). For oddurensinoid **B**, the molecular formula was determined as C_30_H_50_O_2_ based on high-resolution mass spectrometry (HRMS), which gave an [M]^−^ ion at *m*/*z* 441.37 (calculated molecular mass for C_30_H_50_O_2_: 442.7, Supporting Information, Table S1, and Figure S2). In the IR spectrum (Supporting Information, Figure S1), oddurensinoid **B** exhibited a broad absorption band at 3433 cm^–1^, characteristic of hydroxyl group stretching. This band appears at a lower frequency than that of the typical free hydroxyl group band (∼3650 cm^–1^). This is likely due to the formation of hydrogen bond between the hydroxyl groups, which may happen either intramolecularly or intermolecularly, depending on the relative position and orientation of the hydroxyl groups. Additionally, a medium to weak absorption band at 1642 cm^–1^ was observed, corresponding to C=C stretching vibrations, indicating the presence of at least one olefinic bond within the structure.

**Figure 1:**
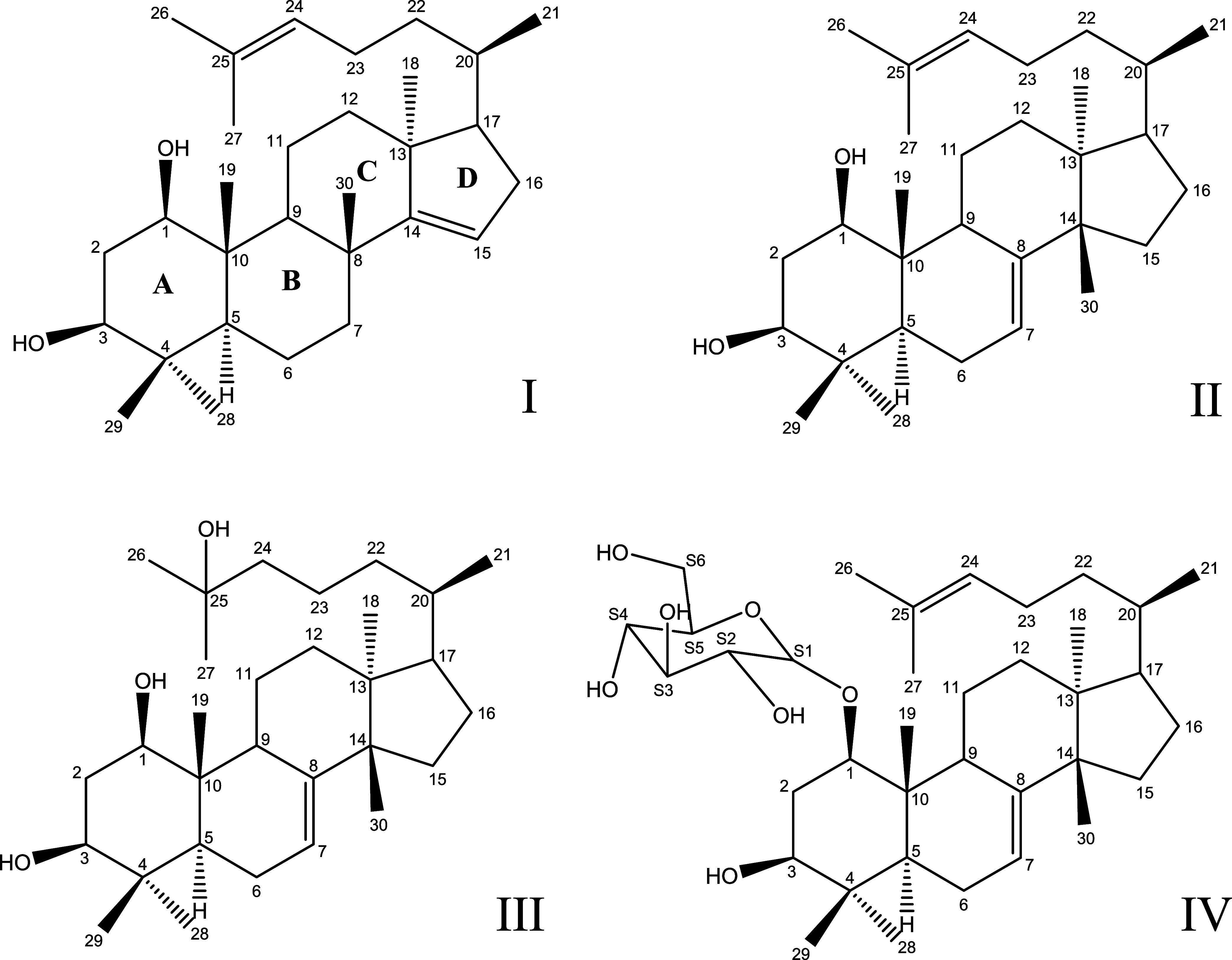
Structure and numbering of the atoms for compounds myrrhasin (I), oddurensinoid **B** (II), oddurensinoid **H** (III), and oddurensinoid **K** (IV). The original notations at 18 and 30 in myrrhasin (I) are switched from the literature reference to facilitate fast and consistent comparison between myrrhasin and oddurensinoid compounds.

Oddurensinoid **B** is the first of the three compounds that we elucidated during the analysis. The 1D ^13^C NMR spectrum, together with DEPT-135 and DEPT-90 experiments (Supporting Information, Figures S5, S10, and S11), revealed a total of 30 carbon resonances, which were classified as follows: eight methyls, assigned to C-18, C-19, C-21, C-26, C-27, C-28, C-29, and C-30; eight methylenes, assigned to C-2, C-6, C-11, C-12, C-15, C-16, C-22, and C-23; eight methines, including two oxygenated methine (C-1 and C-3), two olefinic methines (C-7 and C-24), and four aliphatic methines (C-5, C-9, C-19, and C-20); six quaternary carbons, comprising four aliphatic quaternaries (C-4, C-10, C-13, and C-14), and two olefinic quaternaries (C-8 and C-25). The proton and carbon chemical shifts are located through 1D ^1^H NMR, 2D ^1^H–^13^C HSQC, and 2D ^1^H *J*-resolved NMR. The combined carbon skeleton accounts for a formula of C_30_H_48_, which, together with two additional hydroxyl groups (observed in IR and HRMS, Supporting Information, Figures S1–S2), yields the molecular formula C_30_H_50_O_2_. This composition corresponds to six degrees of unsaturation, consistent with a tetracyclic triterpene bearing two double bonds. The spectroscopic data described above strongly suggest that oddurensinoid **B** is a tetracyclic triterpenoid diol bearing two carbon–carbon double bonds. This structural framework aligns closely with previously reported euphane-type triterpenoid diols such as compound **1** from *Garuga pinnata* ^25^ and myrrhasin from *Commiphora myrrha*,^24^ both of which share the same molecular formula (C_30_H_50_O_2_). While the ^1^H NMR data of compound **1** from *Garuga pinnata* ^25^ are only partially assigned, its complete ^13^C NMR data offer a valuable basis for comparison. A detailed examination reveals that the ^13^C NMR chemical shifts for oddurensinoid **B** closely match those of the euphane triterpenoid compound **1** from *Garuga pinnata* (Supporting Information, Table S2), supporting the hypothesis that they share a common euphane-type skeleton. This structural similarity justified further comparative analysis to confirm connectivity and substitution patterns, which ultimately aided in the structure elucidation of oddurensinoid **B**.

The core skeleton for oddurensinoid **B** was mapped out using 2D NMR techniques including COSY, TOCSY, and HSQC-TOCSY correlations (Figure 2). Ring A, comprising C-1, C-2, C-3, C-4, C-5, C-10, and C-19, shows strong spectral similarity to compound **1** from *Garuga pinnata*, supporting a shared euphane-type A-ring framework. The COSY and HSQC-TOCSY spectra (Figure 2) clearly establish a spin system between C-1/H-1, C-2/H-2, and C-3/H-3.Notably, the oxygenated methines at C-1 and C-3 exhibit downfield ^13^C chemical shifts of 76.54 and 75.91 ppm, respectively, while their corresponding protons resonate at 3.55 ppm­(H-1) and 3.30 ppm (H-3). These shifts are consistent with hydroxyl substitution. HMBC correlations enable clear differentiation between H-1 and H-3: H-1 shows correlation with a single methyl group (Me-19, δ_C_ = 7.48 ppm), which is notably upfield shifted, likely due to the γ-gauche effect of the hydroxyl group at C-1.^26^ H-3 correlates with two methyl groups, Me-28 (δ_C_ = 27.19 ppm) and Me-29 (δ_C_ = 14.13 ppm), which are themselves intercorrelated in the HMBC spectrum and have chemical shifts closely resembling those observed in myrrhasin, indicating a conserved ring architecture.

**Figure 2:**
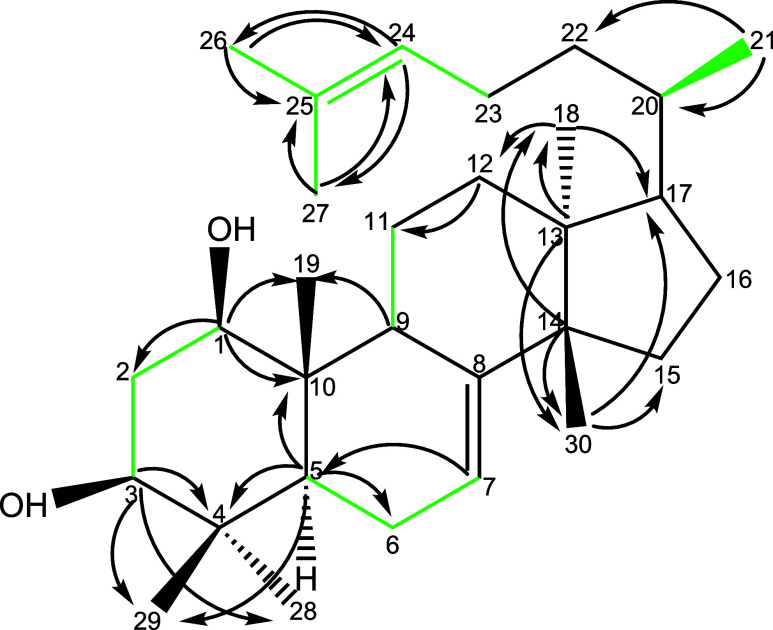
COSY and ^1^H–^13^C HMBC correlations of the compound oddurensinoid **B**. Green color indicates the observation of attached proton bond correlation in the COSY spectroscopy. Pointed arrow indicates the observation of attached proton correlation with carbon of pointed arrow in 2D ^1^H–^13^C HMBC spectroscopy.

The ^1^H NMR multiplicities and coupling constants further support the stereochemistry: H-1 appears as a doublet of doublets (dd, *J* = 11.6, 4.6 Hz). H-3 also appears as a doublet of doublets (dd, *J* = 12.3, 3.8 Hz). The large coupling constants (∼11–12 Hz) are indicative of axial–axial relationships, suggesting that both hydroxyl groups at C-1 and C-3 are β-oriented. The two diastereotopic H-2 protons appear at 1.92 ppm (H-2_α_) and 1.68 ppm (H-2_β_), with an overlapping multiplet in the 1D ^1^H spectrum. Analysis via 2D ^1^H *J*-resolved NMR (Supporting Information, Figure S6) resolved both into “ddd” patterns with a geminal coupling of approximately 12 Hz, confirming their relationship with H-1 and H-3. H-2_α_ proton (1.92 ppm) shows small coupling (∼4.6 Hz to H1; ∼3.8 Hz to H3), consistent with β-orientation. H-2_β_ (1.68 ppm) exhibits strong couplings (∼11.6 Hz to H-1; ∼12.3 Hz to H-3), suggesting an α orientation, placed on the opposite face of ring A relative to H-2_α_. These observations imply a dihedral angle of nearly 180° between H-2_β_ and both H-1 and H-3, reinforcing their axial alignment. To further support spatial interpretation (Figure 3), a 3D molecular model was constructed using Chem3D with an MMFF94 force field and energy minimization. This model helps visualize spatial relationships and coupling effects. It also explains why H-2_α_ exhibits weaker scalar coupling but stronger NOE correlations than H-2_β_ (Figure 4). NOESY data provides additional stereochemical conformation: A strong NOESY cross-peak between H-3 and H-5 (Figure 3) supports an α orientation for H-5. Me-28 shows stronger NOESY with H-3 than Me-29, suggesting Me-28 is α-oriented, while Me-29 is β-oriented. The NOESY cross-peak intensity between Me-19 and H-1 has a similar weak intensity as that of Me-29 and H-3, suggesting that Me-19 takes β orientations. Me-19, being further away from H-5, shows minimal NOESY, as expected. Altogether, these NMR data and spatial relationships strongly support the relative configuration of ring A, including the β-orientation of hydroxyl groups at C-1 and C-3, and confirm the euphane-type triterpenoid diol structure of oddurensinoid **B**.

**Figure 3:**
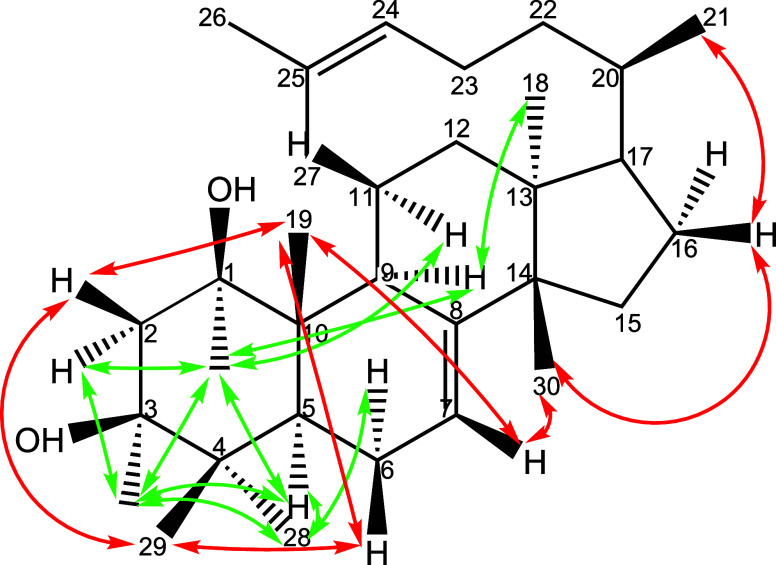
Selected NOESY correlations of compound oddurensinoid **B**. Red arrows connect proton resonances favoring β orientations. Green colored arrows connect proton resonances favoring α orientations.

**Figure 4:**
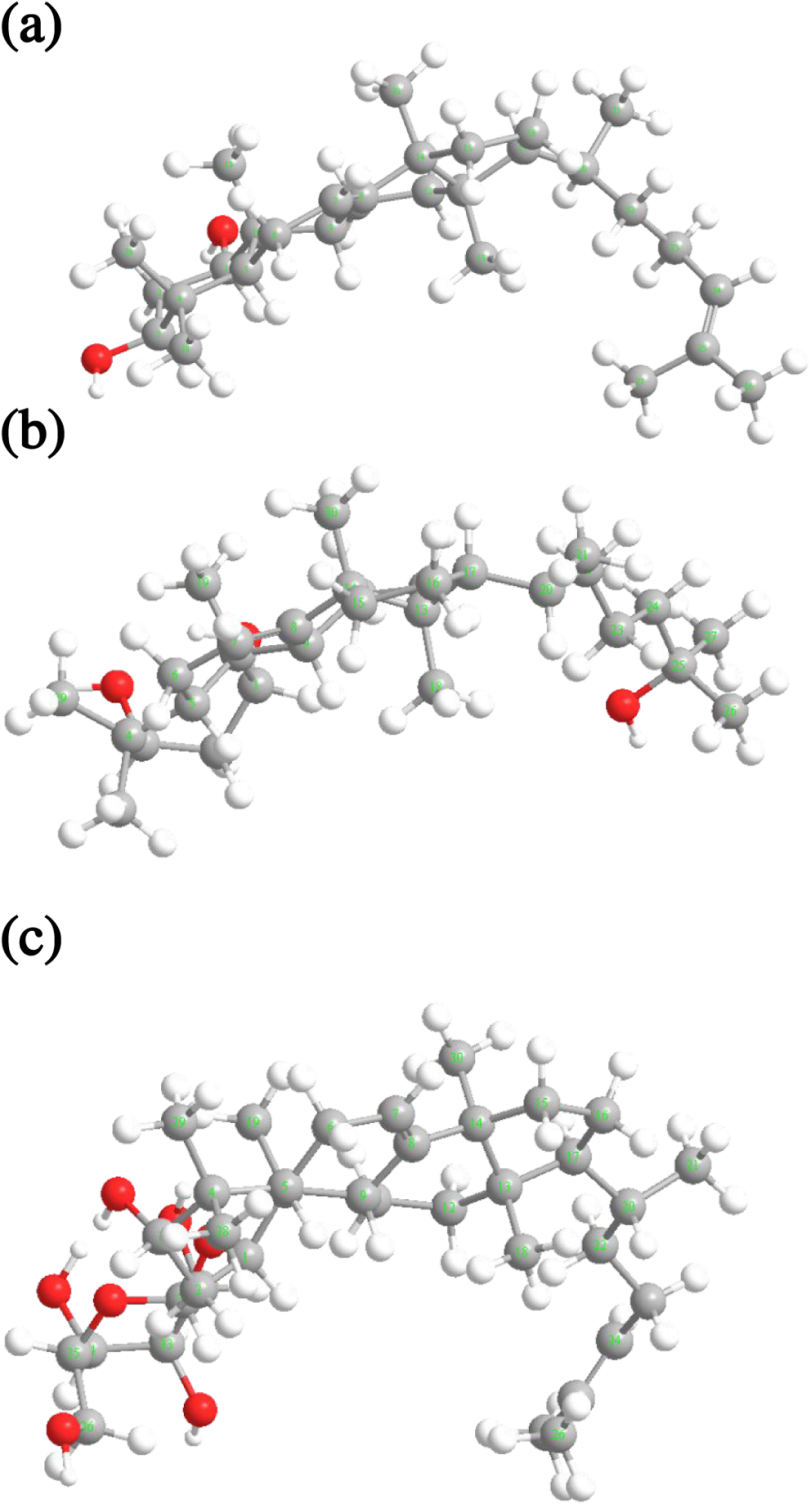
Energy-minimized 3D model structures for oddurensinoid **B** (a), oddurensinoid **H** (b), and oddurensinoid **K** (c). Chem3D program and MMFF94 force field are utilized for energy minimization. Carbon atoms 31–36 in oddurensinoid **K** correspond to S1–S6 for the hexose moiety.

The spin system encompassing C-5, C-6, C-7, and C-9 was established via HSQC-TOCSY and COSY correlations (Figure 2), confirming their connectivity within ring B, which contains a characteristic C=C double bond. The carbon chemical shifts in this region closely mirror those reported for compound 1 from *Garuga pinnata*,^25^ further supporting a shared euphane-type skeleton. The olefinic proton H-7 resonates at 5.26 ppm, and the associated C-7 carbon signal appears at 117.71 ppm, both showing downfield shifts consistent with a C7=C8 double bond. The adjacent C-8 carbon, highly deshielded due to its sp^2^ hybridization, appears at 145.86 ppm—the most downfield carbon signal in the spectrum, and is correlated to C-7 in the HMBC spectrum. The multiplet pattern for H-7 is observed as a doublet of doublets (dd), with small coupling constants of approximately 3 and 1 Hz, indicative of equatorial or β-orientation. This stereochemical assignment is further supported by a strong NOESY cross-peak between H-7 and Me-19, suggesting their spatial proximity. In contrast, H-9 appears at 2.36 ppm and shows NOESY correlations with both H-1 and H-5, suggesting that H-9 takes an α orientation (Figures 3 and 4). These interactions, when considered alongside the relative spatial arrangement predicted from the euphane framework, support the α-orientation of H-9. Altogether, these data confirm the presence of a double bond between C-7 and C-8, and support the β-orientation of H-7 and α-orientation of H-9, thereby helping to define the stereochemistry and substitution pattern of ring B in oddurensinoid **B**.

Additional insights into ring C were obtained through extended HSQC-TOCSY analysis, which successfully traced the C-9–C-11–C-12 spin system, facilitating the assembly of the C-9 to C-12 fragment. Within this region, two unassigned quaternary carbon signals in the aliphatic region appeared at 43.02 and 51.16 ppm, respectively. Their chemical shifts and similar HMBC correlation patterns suggested proximity and potential assignment of C-13 and C-14. To support these assignments, the ChemDraw NMR prediction tool was employed. It estimated a chemical shift of ∼ -43.5 ppm for C-13, and ∼51.3 ppm for C-14, aligning closely with the observed data. Based on this, the signal at 43.02 ppm was confidently assigned to C-13, and that at 51.16 ppm to C-14—an assignment later corroborated by further NMR correlations.

Apart from the overlapping resonance of C-11, the rest of the ring C carbon and proton assignments aligned well with the data reported for compound 1 from *Garuga pinnata*.^25^ The severe overlap of the C-11 methylene protons posed a challenge in direct 1D assignments; however, this was overcome through HSQC-TOCSY, which enabled resolution and definitive assignment of C-11/H-11. Two previously unassigned methyl groups, observed at 0.96 and 0.78 ppm, respectively, exhibited HMBC cross-peaks with C-12, C-13, and C-14. The methyl resonance at 0.96 ppm additionally showed a distinct HMBC correlation with C-8, suggesting its identity as Me-30, this assignment is justified by the stronger expected ^3^ *J* coupling of Me-30 to C-8 than ^4^ *J* between Me-18 to C-8. In the NOESY spectrum, Me-30 displayed a strong spatial correlation with H-7, but it lacked correlation with H-9, supporting its β-orientation. Conversely, the methyl group at 0.78 ppm, assigned as Me-18, showed a strong NOESY cross-peak with H-9, indicating its α-orientation (Figures 3 and 4). These spatial proximities help refine the stereochemical arrangement within ring C and further validate the methyl group orientations.

Further examination of the 2D ^1^H–^13^C HMBC spectrum revealed Me-30s correlation with C-15, while Me-18, resonating at 0.78 ppm, showed HMBC connectivity to C-12. Although C-15 (34.21 ppm) and C-12 (34.02 ppm) are close in chemical shift, they could be unambiguously distinguished by their respective long-range correlations. This spectral differentiation firmly supports the current methyl group assignments, which notably differ from those reported for compound 1 from *Garuga pinnata*. We attribute the increased confidence in these assignments to two critical advantages: (1) the use of a higher-field NMR instrument and (2) the inclusion of HSQC-TOCSY spectra, which provided more detailed cross-peak information. In crowded aliphatic regions, 1D ^1^H–^1^H TOCSY spectra often suffer from spectral overlap. In contrast, ^1^H–^13^C HSQC-TOCSY offers superior spectral dispersion due to the broader chemical shift range of carbon, allowing for clearer visualization and assignment of coupled systems. Additionally, the 2D ^1^H *J*-resolved spectrum (Supporting Information, Figure S6) showed that both Me-18 and Me-30 appear as small doublets, with coupling constants of approximately 1.2 Hz, suggesting the presence of long-range (^4^ *J*) coupling, likely with protons on C-12 and C-15, respectively. These doublets are not readily resolved in conventional 1D ^1^H NMR spectra due to their small *J* values and inherent line width. In contrast, the remaining five methyl groups—Me-19, Me-26, Me-27, Me-28, and Me-29—are attached to quaternary carbons and consistently appear as singlets, as expected. This consistent observation of small methyl doublets for Me-18 and Me-30 was also noted in the two other newly identified compounds, oddurensinoid **H** and **K**, isolated from *Commiphora oddurensis*. This reproducible pattern provides an effective diagnostic feature that may facilitate faster and more reliable methyl group assignments in the structure elucidation of other structurally related tirucallane-type triterpenoids in the future.

As expected, a COSY correlation between H-20 and Me-21 was observed, indicating a scalar coupling between these protons. In the HSQC-TOCSY spectrum, the C-17 carbon resonance at 53.21 ppm showed connectivity with protons corresponding to C-15 and C-16, establishing a continuous spin system: CH_2_–15 → CH_2_–16 → CH-17 → CH-20 → Me-21. These correlations confidently complete the assignments for this segment of the molecule. NOESY cross-peaks provided crucial spatial orientation information: strong NOESY correlations between H-16 and both Me-30 and Me-21 suggest that Me-21 adopts a β-orientation. This stereochemical assignment contrasts with that reported for compound 1 from *Garuga pinnata*,^25^ where Me-21 was proposed to be α-oriented. This discrepancy indicates that oddurensinoid **B** may represent either a structurally distinct new compound or a diastereomer of the previously reported structure. Further HSQC-TOCSY analysis revealed a CH_2_–CH_2_–CH spin system, featuring a downfield-shifted methine proton at δ_H_ = 5.09 ppm and a carbon at δ_C_ = 125.10 ppm. These shifts are characteristic of olefinic protons, supporting assignment to the C-22 to C-24 region, which contains a C24–C25 double bond. The adjacent quaternary carbon C-25 was readily identified at δ_C_ = 130.97 ppm based on its chemical shift and HMBC correlations. Two methyl groups, Me-26 and Me-27, exhibited clear HMBC correlations with both C-24 and C-25, enabling their confident assignment. Notably, the only methyl group coupled to a methine carbon (C-20) was observed at δ_H_ = 0.85 ppm, appearing as a doublet with a coupling constant of ∼6.6 Hz (Supporting Information, Figure S6). This pattern confirms its identity as Me-21, consistent with it being attached to C-20, the only methine carbon bearing a methyl substituent. HMBC correlations of Me-21 with both C-17 and C-22 further reinforce this assignment.

During the isolation of the three triterpenoids described in this study, we also isolated myrrhasin, or 30(14–13) abeo-dammara-14,24-diene-1β,3β-diol, from *Commiphora oddurensis*. The structure of myrrhasin has been previously reported^24^ and included in Figure 1 for reference. Myrrhasin shares a high degree of structural similarity with oddurensinoid **B**, particularly in its stereochemical configuration. Notably, the NOESY correlations observed in oddurensinoid **B** closely mirror those seen in myrrhasin. Specifically, cross-peaks between H-1, H-3, H-5, H-9, and Me-19, Me-28, Me-29 are consistent with previously reported NOE patterns in myrrhasin. These correlations support the assignment of β-orientation for the hydroxyl groups at C-1 and C-3, as well as for Me-19, Me-29, Me-30, and Me-21. Conversely, H-1, H-3, H-5, and H-9, along with Me-18 and Me-28, are inferred to adopt an α-orientation (Figures 3 and 4). These observations reinforce the stereochemical assignments in oddurensinoid **B** and further validate its structural relationship to myrrhasin while also highlighting subtle but meaningful differences between the two compounds.

Comparing the stereochemical orientation of oddurensinoid **B** with reported euphane and lanostane compounds^25,27^ reveals that all three share the same 3β and 9β orientations. However, notable differences arise in the orientations of the methyl groups at positions C-18, C-21, and C-30. In tirucallane-type compounds, Me-30, Me-21 typically adopt β-orientations, while Me-18 is α-oriented. In contrast, lanostane-type compounds exhibit the reverse: Me-30 and Me-21 adopt α orientations, and Me-18 is β-oriented. The orientation pattern observed in oddurensinoid **B** clearly matches that of tirucallane-type triterpenoids. This assignment is strongly supported by the NOESY correlations (Figure 3): H-9 shows strong NOESY cross-peaks with H-1, H-3, and H-5, all of which are α-oriented. Additionally, H-9 exhibits a strong NOESY correlation with Me-18 but not with Me-30, consistent with Me-18 being α-oriented and Me-30 being β-oriented. Further, NOESY cross-peaks between Me-19 and Me-30, and between Me-30 and Me-21, support this stereochemical configuration. Based on these observations, we conclude that oddurensinoid **B** adopts the tirucallane skeleton and shares the characteristic stereochemistry of this class. Accordingly, we propose the full name of this compound as tirucalla-7,24-diene-1β,3β-diol.

For oddurensinoid **K**, the molecular formula was established as C_36_H_60_O_7_ based on high-resolution mass spectrometry (HRMS), which showed a molecular ion peak at *m*/*z* 627.42 [M + Na]^+^, consistent with the calculated mass (calculated molecular mass for C_36_H_60_O_7_: 604.9, Supporting Information, Table S1, Figure S3). This report marks the first structural elucidation and documentation of oddurensinoid **K**. Analysis of the 1D ^13^C NMR, DEPT-135, and DEPT-90 spectra (Supporting Information, Figures S7, S14, and S15) confirms the presence of 36 carbon atoms, including: eight methyl carbons: C-18, C-19, C-21, C-26, C-27, C-28, C-29, and C-30; nine methylene carbons: C-2, C-6, C-11, C-12, C-15, C-16, C-22, C-23, and S-6; 13 methine carbons: including oxygenated methines at C-1 and C-3; olefinic methines at C-7 and C-24; and saturated methines at C-5, C-9, C-17, C-20, and S-1 to S-5; six quaternary carbons: C-4, C-10, C-13, and C-14 (saturated); and C-8 and C-25 (olefinic). Proton chemical shifts were assigned based on 1D ^1^H NMR, 2D ^1^H–^13^C HSQC, and 2D ^1^H *J*-resolved NMR experiments. Complete NMR assignments are presented in Table S4 (Supporting Information). Due to the limited solubility of oddurensinoid **K** in CDCl_3_, additional NMR experiments were conducted in methanol-d_4_, and the corresponding chemical shift assignments are provided in Table S5 (Supporting Information). Both data sets were used for spectral analysis, but chemical shifts obtained from CDCl3 are referenced in Section 3. Most NMR resonances and their assignments in oddurensinoid **K** were achieved by superimposing its spectra with those of oddurensinoid **B**, due to the high structural similarity between the two compounds. The primary distinction lies in the glycosylation at the C-1 position in oddurensinoid **K**. This modification leads to notable chemical shift perturbations at both C-1 and C-2, consistent with typical deshielding effects observed upon C-1 hydroxyl glycosylation. In the ^13^C NMR spectrum, four newly appearing resonances at around 70 ppm are attributed to the hexose moiety. The C-1 and C-3 assignments in oddurensinoid **K** were made using the well-resolved H-1 signal at 3.60 ppm and the H-3 signal at 3.24 ppm. The proton and carbon assignments for the hexose unit were established through a combination of HSQC-TOCSY, TOCSY, COSY, HSQC, and 1D ^1^H NMR data (Supporting Information, Figure S8). A seven-proton spin system was clearly identified in the TOCSY spectrum, and the COSY correlations (Figure 5) supported the sequential connectivity of S-1 through S-6, with S-6 corresponding to the terminal methylene group. The anomeric carbon (S-1) displayed a characteristic chemical shift at 99.30 ppm, aiding its unambiguous identification. The NMR assignment of glycosylated triterpenoids has been previously reported.^28,29^ In agreement with established chemical shift trends for hexose units in ^13^C NMR,^30^ the anomeric proton signal at 4.58 ppm supports the presence of a β-configuration for the sugar moiety. The site of glycosylation was confirmed to be C-1, rather than C-3, based on the observed HMBC cross-peaks between C­(S-1) and H-1, and between C-1 and H­(S-1) (Supporting Information, Figure S9). Based on these data, the sugar moiety is assigned as a β-d-glucopyranoside, and the complete structure of oddurensinoid **K** is thus determined to be tirucalla-7,24-diene-3β-ol-1-*O*-β-d-glucopyranoside. A 3D molecular model was constructed using Chem3D with the MMFF94 force field and energy minimization and is shown in Figure 4b to help visualize the spatial relationships.

**Figure 5:**
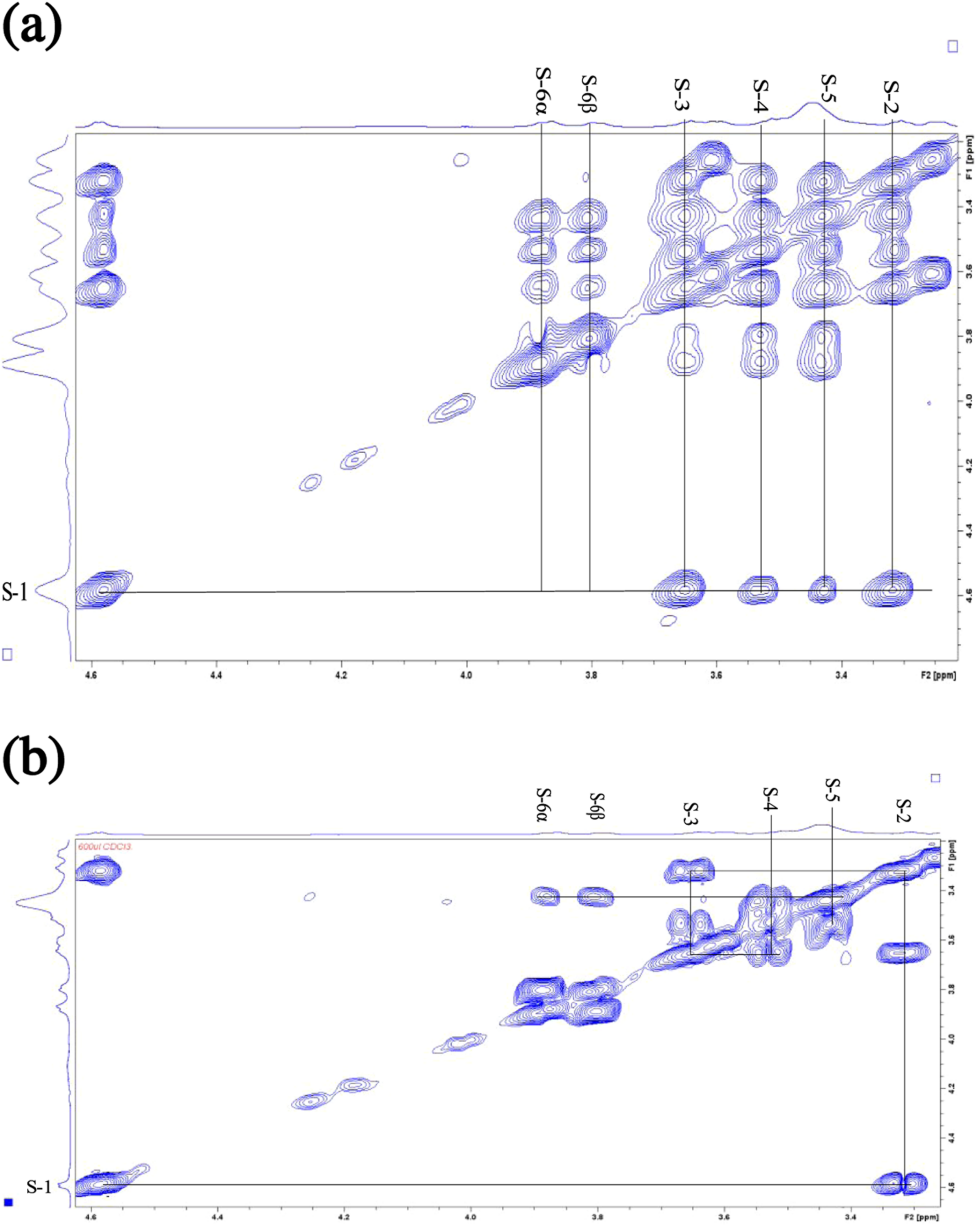
Selected plots of TOCSY spectrum (a) and COSY spectrum (b) showing the connectivity of proton resonances from hexose in oddurensinoid **K**. The NMR spectrometer has an operating frequency of 599.84 MHz for ^1^H and 150.83 MHz for ^13^C. CDCl3 was used as the solvent.

The molecular formula of oddurensinoid **H** was determined to be C_30_H_52_O_3_ based on high-resolution mass spectrometry (HRMS), with the observed *m*/*z* for [M]^−^ consistent with the calculated value (Calcd:459.38) (calculated molecular mass for C_30_H_52_O_3_: 460.7, Supporting Information, Table S1, Figure S4). The combined analysis of the ^13^C NMR, DEPT-135, and DEPT-90 spectra revealed the presence of 30 carbon atoms, categorized as follows: eight methyl groups (C-18, C-19, C-21, C-26, C-27, C-28, C-29, and C-30), nine methylene carbons (C-2, C-6, C-11, C-12, C-15, C-16, C-22, C-23, and C-24), seven methine carbons, including two oxygenated methines (C-1 and C-3) and one olefinic carbon (C-7), and six quaternary carbons (C-4, C-8 [olefinic], C-10, C-13, C-14, and C-25 [oxygenated]). Proton chemical shift assignments were made by using 1D ^1^H NMR, 2D ^1^H–^13^C HSQC, and 2D ^1^H *J*-resolved NMR. Full carbon and proton assignments are provided in Table 1. The observed NMR data suggest a molecular framework of C_30_H_49_, with three hydroxyl groups to satisfy the molecular formula C_30_H_52_O_3_. The similarity in carbon and proton chemical shifts, NOESY cross-peaks, and coupling patterns between oddurensinoid **B** and oddurensinoid **H** (Figure 2 vs Figure 6 and Figure 3 vs Figure 7) indicates that the A-ring structure and the orientation of the C-1 and C-3 hydroxyl groups are conserved. Similar spectral patterns observed in rings B–D, including the olefinic bond in ring B, further support structural conservation across the core triterpenoid skeleton.

**Figure 6:**
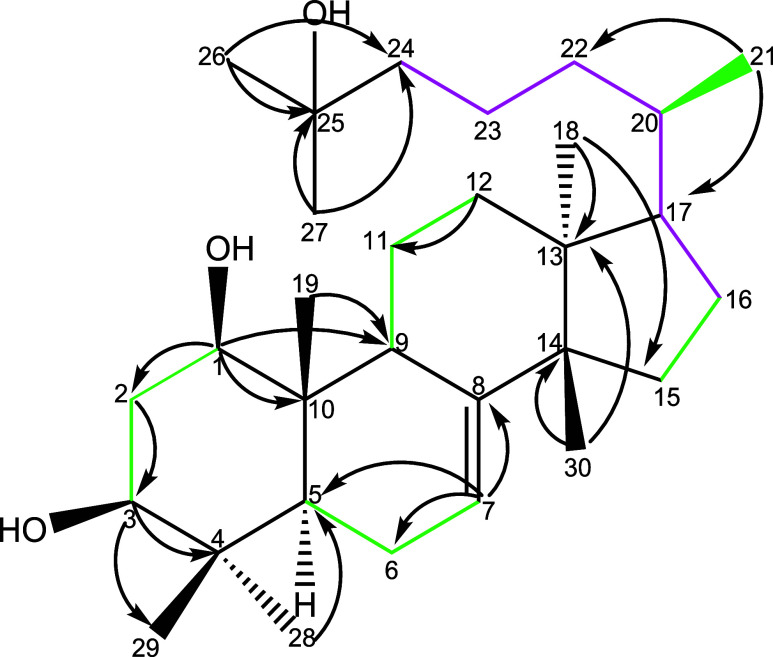
COSY, ^1^H–^13^C HMBC, and HSQC-TOCSY correlations of compound oddurensinoid **H**. Green color indicates the observation of attached proton bond correlation in the COSY spectroscopy. When the COSY correlation is not observed but the HSQC-TOCSY correlation is observed, the correlation is marked in purple color. COSY correlation between H-9 and H-7 is observed but not shown. Pointed arrow indicates the observation of attached proton correlation with carbon of pointed arrow in 2D ^1^H–^13^C HMBC spectroscopy.

**Figure 7:**
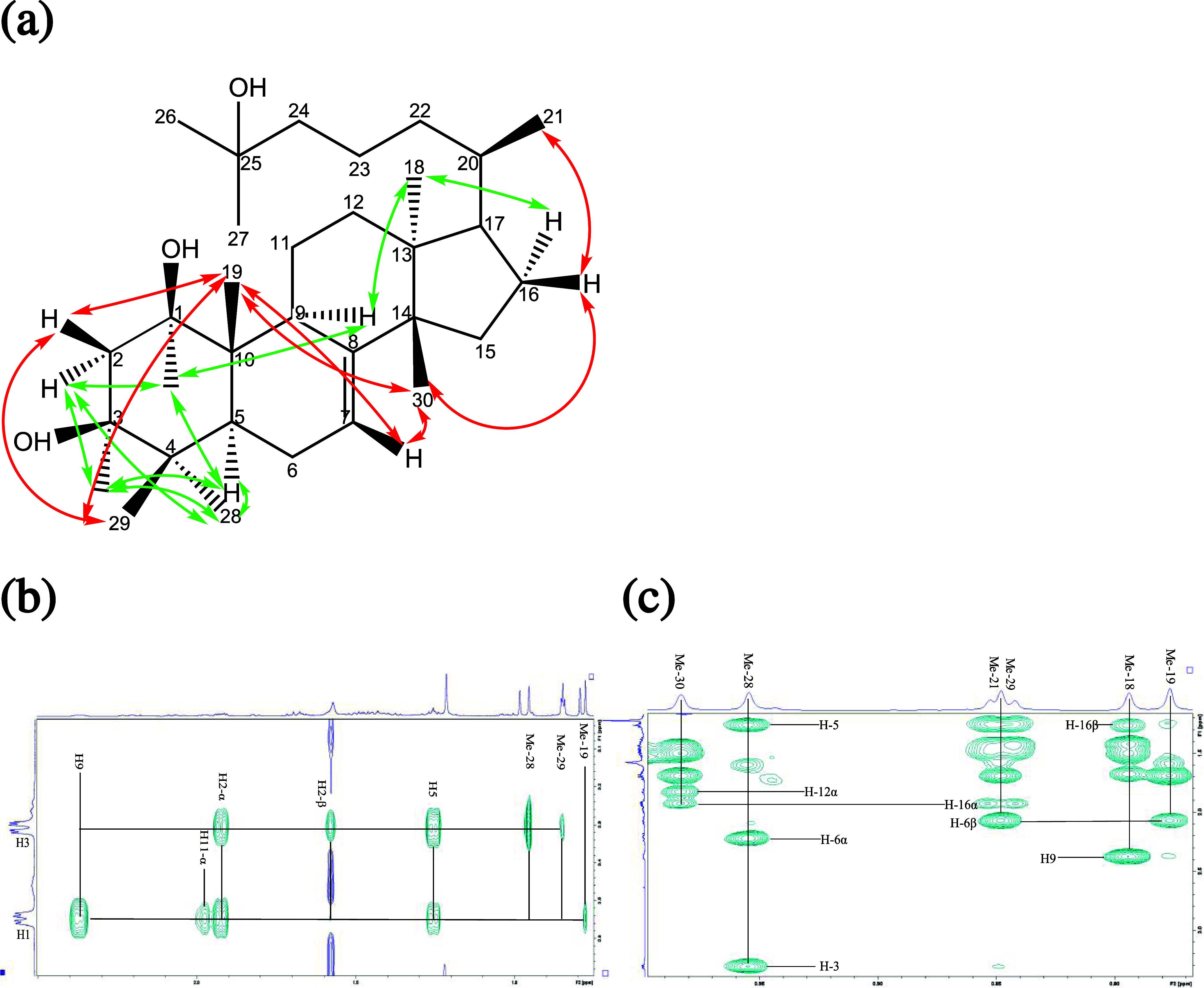
Selected NOESY correlations of compound oddurensinoid **H**. (a) Schematic showing selected NOESY correlations of compound oddurensinoid **H**. Red colored arrows connect proton resonances favoring β orientations. Green colored arrows connect proton resonances favoring α orientations; (b) representative NOESY correlations with proton H-1 and proton H-3; (c) representative NOESY correlations with selected methyl groups.

**Table 1:** Refined ^1^H (600 MHz) and ^13^C (150 MHz) NMR Chemical Shifts Data and Assignments for the Isolated Compound Oddurensinoid H in CDCl_3_ (δ in ppm and *J* in Hz)^a^

oddurensinoid **H**. δ(H) (ppm)		δ(C) (ppm)	COSY	HMBC	HSQC-TOCSY	NOESY
H–C(1)	3.55(dd, *J* = 4.3 Hz, 11.5 Hz)	76.54	2	2_α_,2_β_, 19	1,2,3	2_α_, 3,5,9,19
H_α_–C(2)	1.92(m)(*J*_total_ = 20.8 Hz)	37.95	1,3		1,2,3	1,3
H_β_–C(2)	1.69(m)(*J*_total_ = 36.0 Hz)		1,3			
H–C(3)	3.31(*J* = 3.6 Hz, 12.1 Hz)	75.91	2	2_α_,2_β_, 5,28,29	1,2,3	1, 2_α,_ 5,28,29
C(4)	X	39.03	X	2_α_,2_β_, 5,28,29	X	X
H–C(5)	1.25(m)	49.16	6	19,28,29	5,6,7	1, 6_α_,3,9
H_α_–C(6)	2.24(m)	24.15	5,7	5	5,6,7	5,28
H_β_–C(6)	2.10(m)		5,7			19,29
H–C(7)	5.26(dd, *J* = 3.3 Hz, 1.0 Hz)	117.76	7	6_α_	5,6,7	19,30
C(8)	X	145.83	X	6_α,_6_β_, 11_α_,30	X	X
H–C(9)	2.37(m)	49.56	11	5,19	9,11,12	1,5, 11_α_, 18
C(10)	X	41.07	X	5,19	X	X
H_α_–C(11)	1.98(m)	21.39	9,12		9,11,12	9
H_β_–C(11)	1.68(m)		9,12			
H_α_–C(12)	1.83(m)	33.96	11	18	9,11,12	
H_β_–C(12)	1.67(m)		11			
C(13)	X	43.05	X	18,30	X	X
C(14)	X	51.18	X	15,18,30	X	X
H_α_–C(15)	1.49(m)	34.22	16	30	15,16,17,20,21	
H_β_–C(15)	1.43(m)		16			
H_α_–C(16)	1.92	28.30	15		15,16,17,20,21	
H_β_–C(16)	1.26		15			
H–C(17)	1.51	53.19		18,21	15,16,17,20,21	
Me(18)	0.80(s)	21.98				9
Me(19)	0.78(s)	7.47		5		1, 6_β,_29,30
H–C(20)	1.41(m)	35.91	21	21	15,16,17,20,21	
Me(21)	0.85(d, *J* = 6.3 Hz)	18.59	20		15,16,17,20,21	
					21,22,23,24	
H_α_–C(22)	1.54(m)	35.42	21	21	21,22,23,24	
H_β_–C(22)	1.01(m)					
H_α_–C(23)	1.46(m)	21.27			21,22,23,24	
H_β_–C(23)	1.23(m)					
H_α_–C(24)	1.46(m)	44.27		26,27	21,22,23,24	
H_β_–C(24)	1.38(m)					
C(25)	X	71.12	X	26,27	X	X
Me(26)	1.22(s)	29.32	X	27		
Me(27)	1.22(s)	29.27	X	26		
Me(28)	0.96(s)	27.19	X	29		3, 6_α_
Me(29)	0.85(s)	14.22	X	5,28		3, 6_β,_19
Me(30)	0.98(s)	27.12	X			19

a Chemical shift is referred to as TMS.

Oddurensinoid **H** shows five degrees of unsaturation, one less than oddurensinoid **B**, suggesting the loss of a double bond. The disappearance of the characteristic quaternary carbon resonance (∼130 ppm) and the olefinic methine (∼125 ppm), previously assigned to the C-24–C-25 double bond, indicates a structural alteration in the side chain. As the rest of the structure (rings A and D) remains unchanged, it is plausible that the additional hydroxyl group in oddurensinoid **H** is located on the aliphatic side chain. Detailed COSY and HSQC-TOCSY correlations (Figure 6), particularly between H-16, H-17, H-20, Me-21, H-22, H-23, and H-24, help establish the connectivity of the C-20 to C-24 fragment as CH–CH_2_–CH_2_–CH_2_. The chemical shift of C-24 differs markedly from that of oddurensinoid **B**, consistent with saturation at this position. A new quaternary carbon at δ_C_ 71.12 ppm, likely oxygenated, is assigned as C-25, now bearing a hydroxyl group. This carbon shows HMBC correlations to two methyl groups, consistent with the replacement of Me-26 and Me-27, which now appear upfield shifted due to the loss of conjugation with the former double bond. The introduction of the hydroxyl group provides better chemical shift dispersion in the aliphatic region, aiding in spin system identification.

The relative configuration of oddurensinoid **H** was determined using the same approach as that for oddurensinoid **B**. Key NOESY correlations among H-1, H-3, H-5, H-9, Me-19, Me-28, and Me-29 suggest that the hydroxyl groups at C-1 and C-3, as well as Me-19, Me-29, Me-30, and Me-21, are β-oriented, while H-1, H-3, H-5, H-9, Me-18, and Me-28 are α-oriented (Figures 4 and[Fig fig7]). Based on the above data, the structure of oddurensinoid **H** is assigned as tirucalla-7-ene-1β,3β,25-triol.

The cytotoxic effects of selected isolated compounds were evaluated using the Cell Counting Kit-8 (CCK-8) assay following a 24 h incubation of human cervical cancer (HeLa) cells with varying concentrations of each compound (Figure 8). Among the tested compounds, the newly identified tirucallane-type triterpene, oddurensinoid **H**, exhibited the most potent cytotoxic activity, with an IC_50_ value of 0.017 mg/mL (36.9 μM). This result highlights oddurensinoid **H** as a promising lead candidate for further anticancer drug development. In comparison, glycosylated derivative oddurensinoid **K** displayed moderate activity (IC_50_ = 0.024 mg/mL, 39.7 μM). Oddurensinoid **B** showed the weakest cytotoxicity (IC_50_ = 0.029 mg/mL, 65.5 μM).

**Figure 8:**
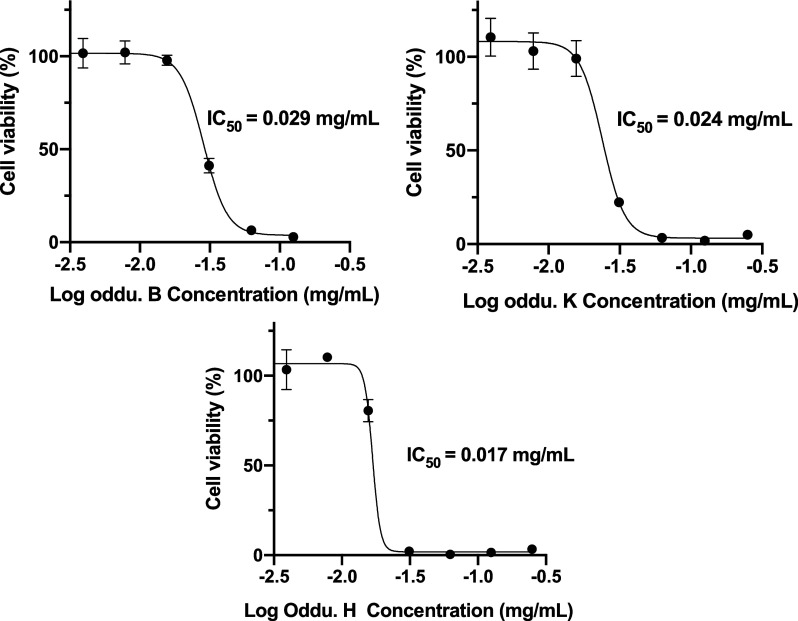
Nonlinear regression curves and the corresponding IC_50_ values. The influence of oddurensinoid **B**, **K**, and **H** is shown on the viability of HeLa cells in the CCK-8 assay.

The structural features of the oddurensinoid compounds appear to play a critical role in their cytotoxic activity against HeLa cells. Notably, oddurensinoid **H**, the most potent compound, contains a tirucallane-type triterpene skeleton with a 1,3-dihydroxyl motif on ring A and an additional hydroxyl group on the side chain. The presence of this side-chain hydroxyl group may enhance hydrogen bonding interactions with molecular targets, potentially increasing cell permeability or binding affinity for proteins involved in cell proliferation regulation. In contrast, oddurensinoid **K** features a similar core structure but is modified by glycosylation, resulting in a hexose moiety attached via an O-glycosidic bond. This structural modification appears to reduce cytotoxic activity, likely due to steric hindrance or altered physicochemical properties such as increased polarity, which may limit membrane permeability or reduce the affinity for target proteins. Furthermore, glycosylation at or near the C-1 position of ring A may disrupt the crucial 1,3-dihydroxyl configuration, a motif that seems to be important for maintaining cytotoxic potency. Oddurensinoid **B**, the least active of the three, retains the triterpene core but lacks both the side-chain hydroxyl group seen in oddurensinoid **H** and the glycosyl modification of oddurensinoid **K**. Its relatively simple hydroxylation pattern suggests that while the base tirucallane scaffold may contribute to some level of cytotoxicity, additional functional groups, especially polar hydroxyls in strategic positions, are essential for optimizing bioactivity. Taken together, these observations suggest that the hydroxylation pattern and glycosylation state significantly influence the cytotoxic potential. The 1,3-dihydroxyl motif on ring A and side-chain hydroxylation appear to be beneficial for anticancer activity, whereas glycosylation may diminish efficacy, possibly by affecting cellular uptake or target engagement. These insights provide a foundation for the rational design and structural optimization of oddurensinoid derivatives as anticancer agents.

## 4 Conclusions

In summary, we have successfully isolated and structurally elucidated three novel tirucallane-type triterpenoids, oddurensinoid **B**, oddurensinoid **H**, and oddurensinoid **K**, from the same plant source. Oddurensinoid **B** has been identified as a diastereomer of compound 1 previously reported from *Garuga pinnata*, while oddurensinoid **H** and oddurensinoid **K** represent newly discovered structures reported here for the first time. All three compounds exhibit cytotoxic activity against human cervical cancer cells (HeLa), with oddurensinoid H showing the most potent effect (IC_50_ = 0.017 mg/mL or 36.9 μM), highlighting its potential as a lead compound for anticancer drug development. The comprehensive NMR-based structure elucidation, supported by multidimensional spectroscopic techniques, including HSQC-TOCSY and NOESY, provides valuable insights that may facilitate the future identification and stereochemical assignment of structurally related triterpenoids. Given the promising biological activity and structural framework, further optimization and derivatization of oddurensinoid **H** may contribute to the development of novel therapeutic agents for cancer treatment.

## Supplementary Material



## Data Availability

NMR data are deposited in the Natural Products Magnetic Resonance Database (NP-MRD) under accession NP0350892 for oddurensinoid **B**, accession NP0350893 for oddurensinoid **H**, and accession NP0350894 for oddurensinoid **K**.

## Notes

UV, IR spectrum of oddurensinoid **B**; mass spectroscopy data of oddurensinoid **B**, **K**, and **H**; detailed NMR chemical shift assignments for oddurensinoid **B** and **K**, and additional NMR spectrum (PDF)

## Abbreviations

DEPT: distortionless enhancement by polarization transfer
HSQC: heteronuclear single quantum coherence
HMBC: heteronuclear multiple bond correlation
COSY: correlation spectroscopy
NOESY: nuclear overhauser enhancement spectroscopy
TOCSY: total correlation spectroscopy
