# Supplementary material for: New Tirucallane-Type Triterpenes Isolated from Commiphora oddurensis from Ethiopia with Anticancer Activity
Source: ACS Omega. 2025 Jul 30;10(31):34559–70. doi: 10.1021/acsomega.5c03203 (PMC12355250; doi:10.1021/acsomega.5c03203)

Supporting Information (SI)

New Tirucallane-type Triterpenes Isolated from *Commiphora oddurensis* from Ethiopia with Anticancer Activity.

Taame G. Seyoum,[a] Zhenming Du,[b*] Siming Wang,[b] Wen Lu,[b] Qiyue Mao,[b] Xiaoxiao Yang,[b] Aman Dekebo,[a] Ermias Dagne,[a] Paulos Yohannes,[c*] Binghe Wang[b]

[a]: Department of Chemistry, Addis Ababa University, Miazia 27 Sq., P.O.Box 30270 Addis Ababa, Ethiopia

[b]: Department of Chemistry, Georgia State University, 50 Decatur St, Atlanta, GA, USA 30303

[c]: Perimeter College, Georgia State University, 2101 Womack Road, Dunwoody, GA, USA, 30338

[*]: Corresponding Authors:

Zhenming Du: Email:zdu@gsu.edu

Paulos Yohannes: Email: pyohannes@gsu.edu

Table of Contents

Table S1. Molecular formula and molecular weight for all three compounds, oddurensinoid **B, H** and **K**.

Table S2. Refined ^{1}H (600 MHz) and ^{13}C (150 MHz) NMR data for oddurensinoid **B** dissolved in $CDCl_3$ (δ in ppm and J in Hz). Trace TMS (0.01%) in $CDCl_3$ is used as chemical shift reference. Oddurensinoid **B** chemical shifts are listed in comparison with data for Euphane triterpenoid compound 1 from *Garuga pinnata*, which is measured in $CDCl_3$, at 300 MHz (^{1}H NMR), 75MHz (^{13}C NMR), using TMS as internal standard.

Table S3. Refined ^{1}H (600 MHz) and ^{13}C (150 MHz) NMR data for oddurensinoid **B** dissolved in $CDCl_3$ (δ in ppm and J in Hz). Trace TMS (0.01%) in $CDCl_3$ is used as chemical shift reference.

Table S4. Refined ^{1}H (600 MHz) and ^{13}C (150 MHz) NMR data for oddurensinoid **B** and oddurensinoid **K** dissolved in $CDCl_3$ (δ in ppm and J in Hz). Trace TMS (0.01%) in $CDCl_3$ is used as chemical shift reference.

Table S5. Refined ^{1}H (600 MHz) and ^{13}C (150 MHz) NMR data for oddurensinoid **K** dissolved in $CDCl_3$ (δ in ppm and J in Hz) and MeOD. Trace TMS (0.01%) in $CDCl_3$ or MeOD is used as chemical shift reference.

Figure S1. UV and IR spectra for oddurensinoid **B.**

Figure S2. Mass Spectrum and results for oddurensinoid **B**.

Figure S3. Mass Spectrum and results for oddurensinoid **K**.

Figure S4. Mass Spectrum and results for oddurensinoid **H**.

Figure S5. DEPT and ^{13}C NMR spectra of oddurensinoid **B**. a) Plot of 1-D ^{13}C NMR spectrum showing the assignments of six quaternary carbon resonances; b) plot of selected region of DEPT 90 spectrum showing the assignments of eight CH groups; c) plot of selected regions of DEPT 135 spectrum showing the assignments of eight CH_2 and eight CH_3 groups.

Figure S6. Selected J-resolved spectra of oddurensinoid **B**. a) Plot of the H-3 and H-3 regions shows the resolved H-3 and H-1 strips that are well resolved; b) plot of the up-field regions that help to resolve the a few important methyl groups; c) plot of extract of H-2_{α} from J-resolved spectrum; d) plot of extract of H-2_{β} from J-resolved spectrum.

Figure S7. Plots of selected regions of ^{13}C NMR spectra of for oddurensinoid **K** with the assignments labelled.

Figure S8. Plots of selected regions of COSY and HMBC spectra for oddurensinoid **K** illustrating the assignments.

Figure S9. Plots of selected regions of HMBC spectra for oddurensinoid **K** elucidating the assignment of hexose connectivity.

Figure S10. Full 1D ^{1}H (600 MHz) NMR spectra of for oddurensinoid **B**.

Figure S11. Full 1D ^{13}C NMR (150 MHz) spectra of for oddurensinoid **B**.

Figure S12. Full 1D ^{1}H (600 MHz) NMR spectra of for oddurensinoid **H**.

Figure S13. Full 1D ^{13}C NMR (150 MHz) spectra of for oddurensinoid **H**.

Figure S14. Full 1D ^{1}H (600 MHz) NMR spectra of for oddurensinoid **K**.

Figure S15. Full 1D ^{13}C NMR (150 MHz) spectra of for oddurensinoid **K**.

Table S1. Molecular formula and molecular weight for all three compounds illustrated, Oddurensinoid **B, H** and **K**.

Compound	Molecular Formula	Molecular Weight/Dalton
oddurensinoid **B**	$C_{30}H_{50}O_2$	442.7
oddurensinoid **K**	$C_{36}H_{60}O_7$	604.9
oddurensinoid **H**	$C_{30}H_{52}O_3$	460.7

Table S2. Refined ^{1}H (600 MHz) and ^{13}C (150 MHz) NMR data for oddurensinoid **B** dissolved in $CDCl_3$ (δ in ppm and J in Hz). Trace TMS (0.01%) in $CDCl_3$ is used as chemical shift reference. Oddurensinoid **B** chemical shifts are listed in comparison with data for Euphane triterpenoid compound 1 from *Garuga pinnata*, which is measured in CDCl3, at 300 MHz (^{1}H NMR), 75MHz (^{13}C NMR), using TMS as internal standard. [&]

oddurensinoid **B**	δ(H)/ppm, and J (Hz)	δ(C)/ppm	compound 1 from *Garuga pinnata*[&] δ(H) /ppm, and J (Hz)[&]	compound 1 from *Garuga pinnata*[&] δ(C)/ppm[&]
H-C(1)	3.55(dd, J=4.6, 11.6 Hz,1H)	76.54	3.55(dd, J=3.8, 12.0 Hz, 1H)	77.4
$H_α$-C(2)	1.92(m,1H)	37.87	1.90(m,1H)	37.8
$H_β$-C(2)	1.68(m,1H)		1.75(m,1H)	
H-C(3)	3.30 (dd, J=3.6, 12.2 Hz,1H)	75.91	3.30 (dd, J=11,4 Hz, 1H)	77.0
C(4)	×	39.01	×	38.9
H-C(5)	1.25(m,1H)	49.15		49.6
$H_α$-C(6)	2.21(m,1H)	24.14		24.1
$H_β$-C(6)	2.06(m,1H)			
H-C(7)	5.26(m,1H)	117.71		117.6
C(8)	×	145.86	×	145.8
H-C(9)	2.36(t,1H)	49.54		49.1
C(10)	×	41.04	×	41.0
$H_α$-C(11)	1.98(m,1H)	21.38		27.0
$H_β$-C(11)	1.68(m,1H)			
$H_α$-C(12)	1.83(m,1H)	34.02*		34.2
$H_β$-C(12)	1.68(m,1H)			
C(13)	×	43.02	×	42.9
C(14)	×	51.16	×	50.9
$H_α$-C(15)	1.49(m,1H)	34.21*		34.0
$H_β$-C(15)	1.43(m,1H)			
$H_α$-C(16)	1.92(m,1H)	28.29		28.0
$H_β$-C(16)	1.27(m,1H)			
H-C(17)	1.49(m,1H)	53.21		52.9
Me18	0.78 (s,3H)	21.96	0.79(s,3H)	14.2
Me19	0.77(s,3H)	7.48	0.77(s,3H)	7.4

H-C(20)	1.39(m,1H)	35.74		35.9
Me21	0.85(d, J=6.6Hz,3H)	18.59	0.88(d, J=6 Hz,3H)	18.2
H_{α}-C(22)	1.58(m,1H)	35.11		36.1
H_{β}-C(22)	0.99(m,1H)			
H_{α}-C(23)	2.03(m,1H)	25.28		24.9
H_{β}-C(23)	1.86(m,1H)			
H-C(24)	5.09(m) (m,1H)	125.10		125.1
C(25)	×	130.97	×	130.8
Me26	1.68 (d, 2.3 Hz,3H)	25.74	1.68(s,3H)	25.6
Me27	1.60 (d, 2.3 Hz,3H)	17.68	1.60(s, 3H)	17.5
Me28	0.94 (s,3H)	27.19	0.95(s,3H)	27.1
Me29	0.84 (s,3H)	14.13	0.84(s,3H)	21.7
Me30	0.96 (s,3H)	27.11	0.97(s,3H)	21.7

*1: C-12 overlaps with C-15. The assignment is confirmed in HMBC through the observation of the cross-peak correlation of C15-Me30, and C12-Me18.

*2: C-15 overlaps with C-12. The assignment is confirmed in HMBC through the observation of the cross-peak correlation of C15-Me30, and C12-Me18.

Reference: [&:]

Venkatraman, G.; Thombare, P. S.; Sabata, B. K. Euphane Triterpenoid from *Garuga pinnata*. *Phytochemistry* **1993**, *32* (1), 161-163.

Table S3. Refined ^{1}H (600 MHz) and ^{13}C (150 MHz) NMR data for oddurensinoid **B** dissolved in $CDCl_3$ (δ in ppm and J in Hz). Trace TMS (0.01%) in $CDCl_3$ is used as chemical shift reference.

oddurensinoid **B**	δ(H)/ppm, and J (Hz)	δ(C)/ppm	COSY	HMBC	HSQC-TOCSY	NOESY
H-C(1)	3.55(dd, J=4.6, 11.6 Hz,1H)	76.54	2_α 2_β	19, 2_α, 2_β	1, 2_α, 2_β ,3	3,5, 9, 2_α,19,29
H_α-C(2)	1.92(m,1H)	37.87	1,3		1, 2_α, 2_β ,3	
H_β-C(2)	1.68(m,1H)		1,3		1, 2_α, 2_β ,3	
H-C(3)	3.30 (dd, J=3.6, 12.2 Hz,1H)	75.91	2_α, 2_β	2_α, 2_β,28 ,29	1, 2_α, 2_β ,3	1, 2_α,5, 28,29,
C(4)	×	39.01	×	28,29	×	×
H-C(5)	1.25(m,1H)	49.15	6_α, 6_β	10,28,29,19	5,6,7	6_α,28
H_α-C(6)	2.21(m,1H)	24.14	5,7		5,6,7	5,28
H_β-C(6)	2.06(m,1H)		5,7		5,6,7	19,29
H-C(7)	5.26(m,1H)	117.71	6_α, 6_β, 9	9	5,6,^{9}H-^{7}C	6_α, 6_β,19,30
C(8)	×	145.86	×	30	×	×
H-C(9)	2.36(t,1H)	49.54	7, 11_α, 11_β	7	9,11,12	1,5,18
C(10)	×	41.04	×	5,19	×	×
H_α-C(11)	1.98(m,1H)	21.38	9		9,11,12	
H_β-C(11)	1.68(m,1H)		9			
H_α-C(12)	1.83(m,1H)	34.02		18	9,11,12	
H_β-C(12)	1.68(m,1H)					
C(13)	×	43.02	×	18,30	×	×
C(14)	×	51.16	×	18,30	×	×
H_α-C(15)	1.49(m,1H)	34.21		30	15,16,17,21	
H_β-C(15)	1.43(m,1H)					
H_α-C(16)	1.92(m,1H)	28.29			15,16,17,21	
H_β-C(16)	1.27(m,1H)					
H-C(17)	1.49(m,1H)	53.21		18,21	15,16,17,21	
Me18	0.78 (s,3H)	21.96		12, 13,14,17		9, 15_β
Me19	0.77(s,3H)	7.48		5,10		30
H-C(20)	1.39(m,1H)	35.74	21	21	20,21,22,23, 24,26,27	
Me21	0.85(d, J=6.6Hz,3H)	18.59	20	17,20,22	20,21,22,23, 24,26,27	
H_α-C(22)	1.58(m,1H)	35.11		21	20,21,22,23, 24,26,27	
H_β-C(22)	0.99(m,1H)					

H_{α}-C(23)	2.03(m,1H)	25.28	24	24,25	20,21,22,23, 24,26,27	
H_{β}-C(23)	1.86(m,1H)		24			
H-C(24)	5.09(m) (m,1H)	125.10	23_{α}, 23_{β} ,26,27	23,26,27	20,21,22,23, 24,26,27	18,23_{α}, 23_{β} ,26,27
C(25)	×	130.97	×	23,26,26	×	×
Me26	1.68 (d, 2.3 Hz,3H)	25.74	24	24,25	20,21,22,23, 24,26,27	24
Me27	1.60 (d, 2.3 Hz,3H)	17.68	24	24,25	20,21,22,23, 24,26,27	
Me28	0.94 (s,3H)	27.19		3,4,5,29		3,5,6_{α}
Me29	0.84 (s,3H)	14.13		3,4,5,28		6_{β}
Me30	0.96 (s,3H)	27.11		8,15,13,14		19

Table S4. Refined ^{1}H (600 MHz) and ^{13}C (150 MHz) NMR data for oddurensinoid **B** and oddurensinoid **K** dissolved in $CDCl_3$ (δ in ppm and J in Hz). Trace TMS (0.01%) in $CDCl_3$ is used as chemical shift reference.

	oddurensinoid **B**		oddurensinoid **K**	
	δ(H)/ppm, and J (Hz)	δ(C)/ppm	δ(H)/ppm, and J (Hz)	δ(C)/ppm
H-C(1)	3.55(dd, J=4.6, 11.6 Hz,1H)	76.54	3.60(m,1H)	82.25
$H_α$-C(2)	1.92(m,1H)	37.87	2.10(m,1H)	32.61
$H_β$-C(2)	1.68(m,1H)		1.67(m,1H)	
H-C(3)	3.30 (dd, J=3.6, 12.2 Hz,1H)	75.91	3.24(m,1H)	75.79
C(4)	×	39.01	×	39.13
H-C(5)	1.25(m,1H)	49.15	1.19(m,1H)	49.10
$H_α$-C(6)	2.21(m,1H)	24.14	2.15(m,1H)	23.80
$H_β$-C(6)	2.06(m,1H)		2.01(m,1H)	
H-C(7)	5.26(m,1H)	117.71	5.26(m,1H)	117.78
C(8)	×	145.86	×	146.40
H-C(9)	2.36(t,1H)	49.54	2.35(m,1H)	49.56
C(10)	×	41.04	×	40.64
$H_α$-C(11)	1.98(m,1H)	21.38	2.35(m,1H)	20.30
$H_β$-C(11)	1.68(m,1H)		1.43(m,1H)	
$H_α$-C(12)	1.83(m,1H)	34.02	1.78(m,1H)	34.50
$H_β$-C(12)	1.68(m,1H)		1.47(m,1H)	
C(13)	×	43.02	×	42.87
C(14)	×	51.16	×	51.26
$H_α$-C(15)	1.49(m,1H)	34.21	1.51(m,1H)	34.50
$H_β$-C(15)	1.43(m,1H)		1.44(m,1H)	
$H_α$-C(16)	1.92(m,1H)	28.29	1.90(m,1H)	28.32
$H_β$-C(16)	1.27(m,1H)		1.25(m,1H)	
H-C(17)	1.49(m,1H)	53.21	1.46(m,1H)	53.39
Me18	0.78 (s,3H)	21.96	0.77(s,3H)	22.48
Me19	0.77(s,3H)	7.48	0.79(s,3H)	8.47
H-C(20)	1.39(m,1H)	35.74	1.38(m,1H)	35.50
Me21	0.85(d, J=6.6Hz,3H)	18.59	0.84(d, J=6.6Hz,3H))	18.54
$H_α$-C(22)	1.58(m,1H)	35.11	1.59(m,1H)	35.12
$H_β$-C(22)	0.99(m,1H)		0.99(m,1H)	
$H_α$-C(23)	2.03(m,1H)	25.28	2.02(m,1H)	25.31
$H_β$-C(23)	1.86(m,1H)		1.89(m,1H)	
H-C(24)	5.09(m,1H)	125.10	5.11(m,1H)	125.11
C(25)	×	130.97	×	130.93

Me26	1.68 (d, 2.3 Hz,3H)	25.74	1.67(s)	25.83
Me27	1.60 (d, 2.3 Hz,3H)	17.68	1.60(s)	17.71
Me28	0.94 (s,3H)	27.19	0.93(s,3H)	27.56
Me29	0.84 (s,3H)	14.13	0.84(s,3H)	14.79
Me30	0.96 (s,3H)	27.11	0.97(s,3H)	27.82
**Sugar**				
H-C(S1)			4.58	99.30
H-C(S2)			3.31	73.77
H-C(S3)			3.65	76.11
H-C(S4)			3.52	71.08
H-C(S5)			3.43	75.36
H_α-C(S6)			3.87	62.72
H_β-C(S6)			3.80	

Table S5. Refined ^1H (600 MHz) and ^{13}C (150 MHz) NMR data for oddurensinoid **K** dissolved in $CDCl_3$ (δ in ppm and J in Hz) and MeOD. Trace TMS (0.01%) in $CDCl_3$ and MeOD is used as ^1H chemical shift reference. Either TMS or indirect reference is used for ^{13}C chemical shift calibration.

	oddurensinoid **K** (in MeOD)		oddurensinoid **K** (in CDCl3)	
	δ(H)/ppm, and J (Hz)	δ(C)/ppm	δ(H)/ppm, and J (Hz)	δ(C)/ppm
H-C(1)	3.83(dd,11.7 Hz, 4.2 Hz, 1H)	79.50	3.60(m,1H)	82.25
$H_α$-C(2)	2.05(m,1H)	31.03	2.10(m,1H)	32.61
$H_β$-C(2)	1.64(m,1H)		1.67(m,1H)	
H-C(3)	3.22 (dd,12.8 Hz, 4.3 Hz,1H)	75.30	3.24(m,1H)	75.79
C(4)	×	38.76	×	39.13
H-C(5)	1.25(m,1H)	49.01	1.19(m,1H)	49.10
$H_α$-C(6)	2.17(m,1H)	23.48	2.15(m,1H)	23.80
$H_β$-C(6)	2.07(m,1H)		2.01(m,1H)	
H-C(7)	5.28(m,1H)	117.69	5.26(m,1H)	117.78
C(8)	×	146.51	×	146.40
H-C(9)	2.44(t,1H)	49.32	2.35(m,1H)	49.56
C(10)	×	40.35	×	40.64
$H_α$-C(11)	2.50(m,1H)	20.36	2.35(m,1H)	20.30
$H_β$-C(11)	1.50(m,1H)		1.43(m,1H)	
$H_α$-C(12)	1.70(m,1H)	34.46	1.78(m,1H)	34.50
$H_β$-C(12)	1.61(m,1H)		1.47(m,1H)	
C(13)	×	42.64	×	42.87
C(14)	×	51.09	×	51.26
$H_α$-C(15)	1.50(m,1H)	34.21	1.51(m,1H)	34.50
$H_β$-C(15)	1.44(m,1H)		1.44(m,1H)	
$H_α$-C(16)	1.93(m,1H)	28.08	1.90(m,1H)	28.32
$H_β$-C(16)	1.26(m,1H)		1.25(m,1H)	
H-C(17)	1.50(m,1H)	53.37	1.46(m,1H)	53.39
Me18	0.82 (s,3H)	21.48	0.77(s,3H)	22.48
Me19	0.85(s,3H)	7.47	0.79(s,3H)	8.47
H-C(20)	1.41(m,1H)	35.59	1.38(m,1H)	35.50
Me21	0.86(d, J=6.6Hz,3H)	17.59	0.84(d, J=6.6Hz,3H))	18.54
$H_α$-C(22)	1.64(m,1H)	35.00	1.59(m,1H)	35.12
$H_β$-C(22)	0.99(m,1H)		0.99(m,1H)	
$H_α$-C(23)	2.04(m,1H)	24.86	2.02(m,1H)	25.31
$H_β$-C(23)	1.90(m,1H)		1.89(m,1H)	
H-C(24)	5.12(m,1H)	124.70	5.11(m,1H)	125.11
C(25)	×	130.39	×	130.93

Me26	1.68 (s,3H)	24.52	1.67(s)	25.83
Me27	1.62 (s,3H)	16.36	1.60(s)	17.71
Me28	0.94 (s,3H)	26.75	0.93(s,3H)	27.56
Me29	0.85 (s,3H)	13.80	0.84(s,3H)	14.79
Me30	1.03 (s,3H)	26.93	0.97(s,3H)	27.82
**Sugar**				
H-C(S1)	4.48	98.19	4.58	99.30
H-C(S2)	3.17	74.08	3.31	73.77
H-C(S3)	3.35	76.86	3.65	76.11
H-C(S4)	3.22	70.96	3.52	71.08
H-C(S5)	3.26	76.67	3.43	75.36
H_α-C(S6)	3.92	62.23	3.87	62.72
H_β-C(S6)	3.65		3.80	

Figure S1. UV and IR spectra for oddurensinoid **B**.

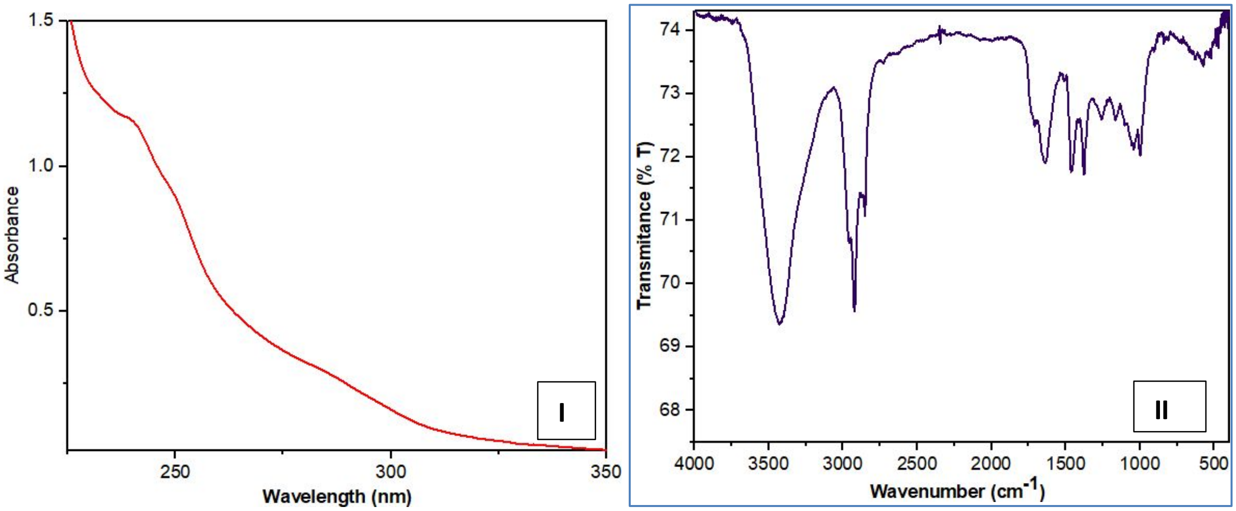

Figure S2. Mass Spectrum and results for oddurensinoid **B**.

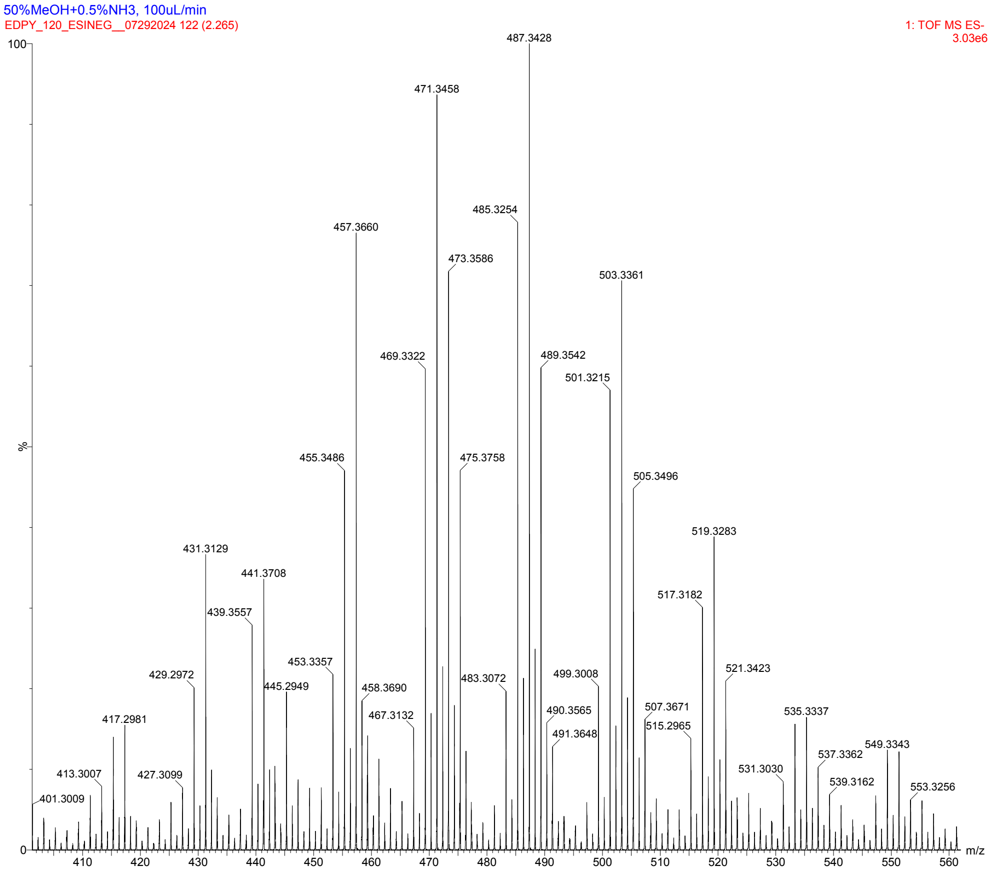

Monoisotopic Mass, Even Electron Ions

729 formula(e) evaluated with 1 results within limits (all results (up to 1000) for each mass)

Elements Used:

C: 30-30 H: 0-101 N: 0-10 O: 0-100 Na: 0-2

Minimum: -50.0

Maximum: 1000.0 20.0 500.0

Mass Calc. Mass mDa PPM DBE Formula

441.3708 441.3733 -2.5 -5.7 6.5 $C_{30} H_{49} O_2$

Mass Spec is run in negative mode, giving a molecular formula of $C_{30}H_{50}O_2$.

Figure S3. Mass Spectrum and results for oddurensinoid **K**.

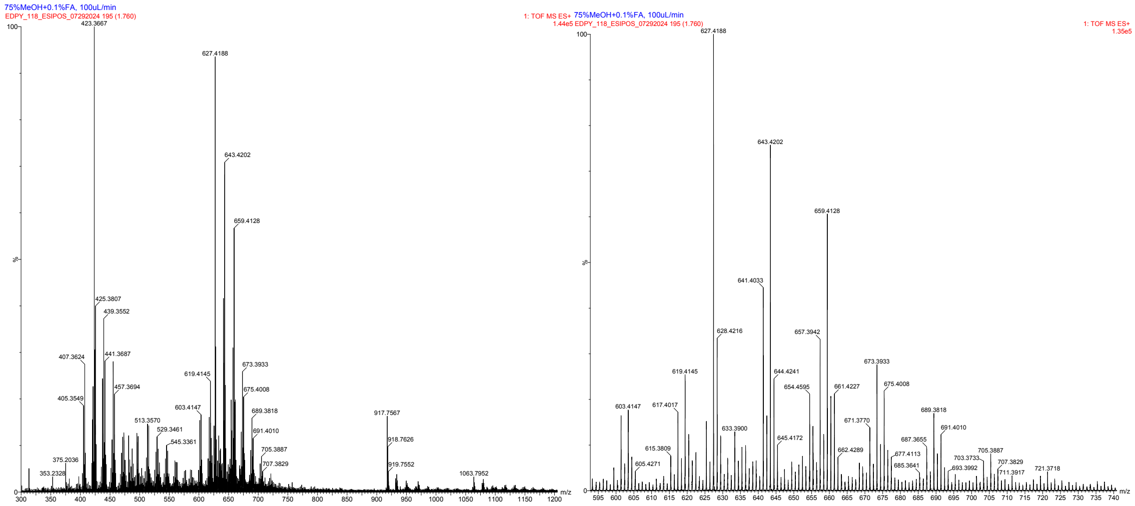

1061 formula(e) evaluated with 3 results within limits (all results (up to 1000) for each mass)

Elements Used:

C: 36-36 H: 0-101 N: 0-10 O: 0-100 Na: 0-2

Minimum:				-50.0		
Maximum:		1000.0		20.0	500.0	
Mass	Calc. Mass	mDa		PPM	DBE	Formula
627.4188	627.4237	-4.9	-7.8	6.5	C36 H60 O7 Na	
	627.4135	5.3	8.4	15.5	C36 H51 N8 O2	
	627.4114	7.4	11.8	8.5	C36 H57 N2 O4 Na2	

Figure S4. Mass Spectrum and results for oddurensinoid **H**.

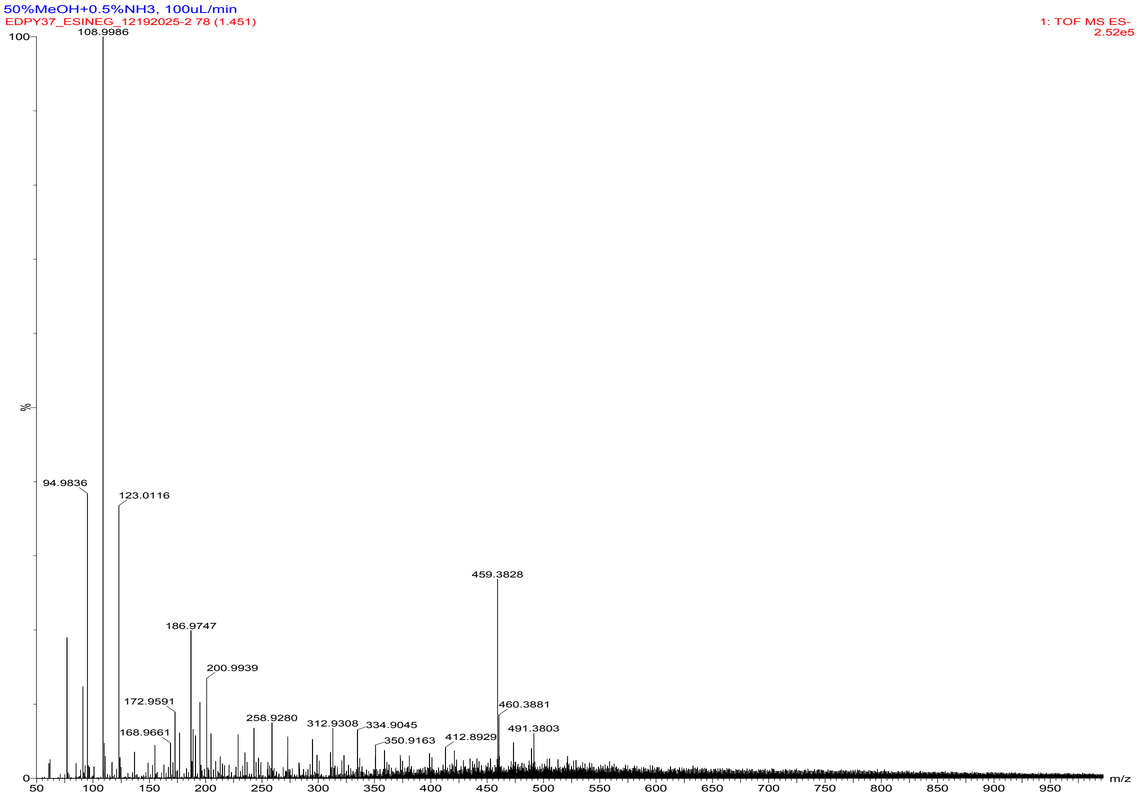

Elements Used:

C: 30-30 H: 0-150 N: 0-7 O: 0-100

Minimum:				-50.0		
Maximum:		1000.0		5.0	500.0	
Mass	Calc. Mass	mDa		PPM	DBE	Formula
459.3828	459.3838	-1.0	-2.2	5.5	C30 H51 O3	

Mass Spec is run in negative mode, giving a molecular formula of $C_{30}H_{52}O_3$.

Figure S5. DEPT and ^{13}C NMR spectra of oddurensinoid **B**. a) Plot of 1-D ^{13}C NMR spectrum showing the assignments of six quaternary carbon resonances; b) plot of selected region of DEPT 90 spectrum showing the assignments of eight CH groups; c) plot of selected regions of DEPT 135 spectrum showing the assignments of eight CH_2 and eight CH_3 groups.

a)

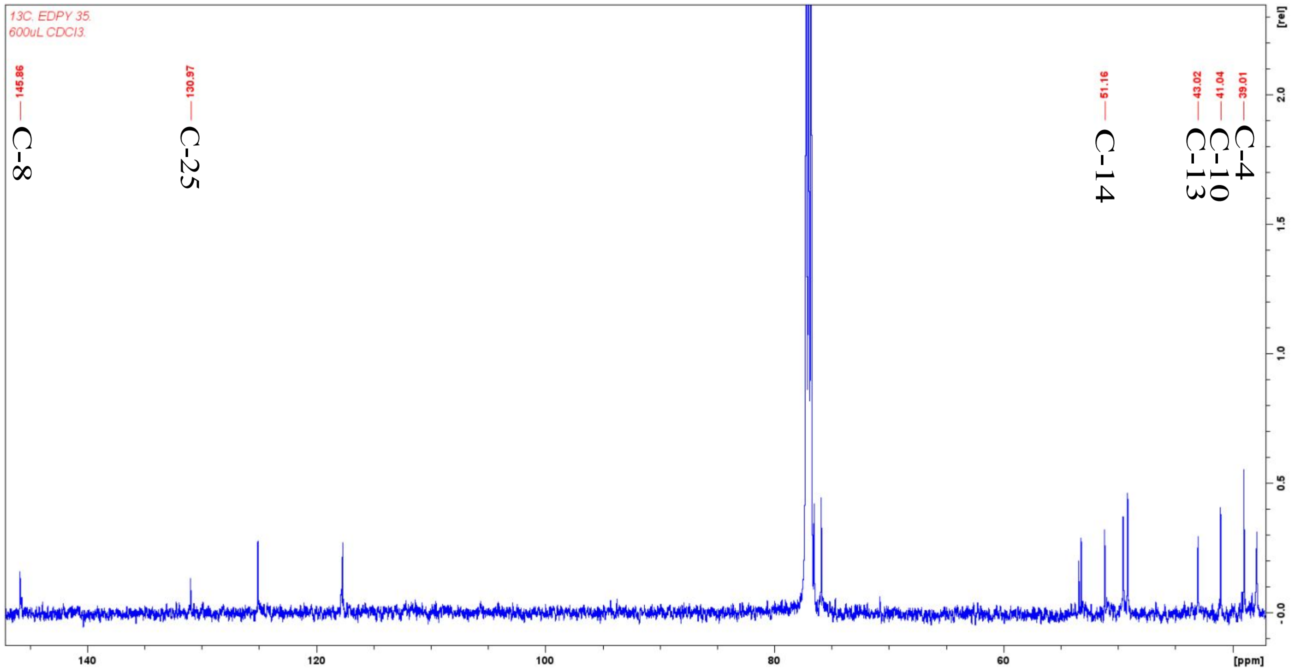

b)

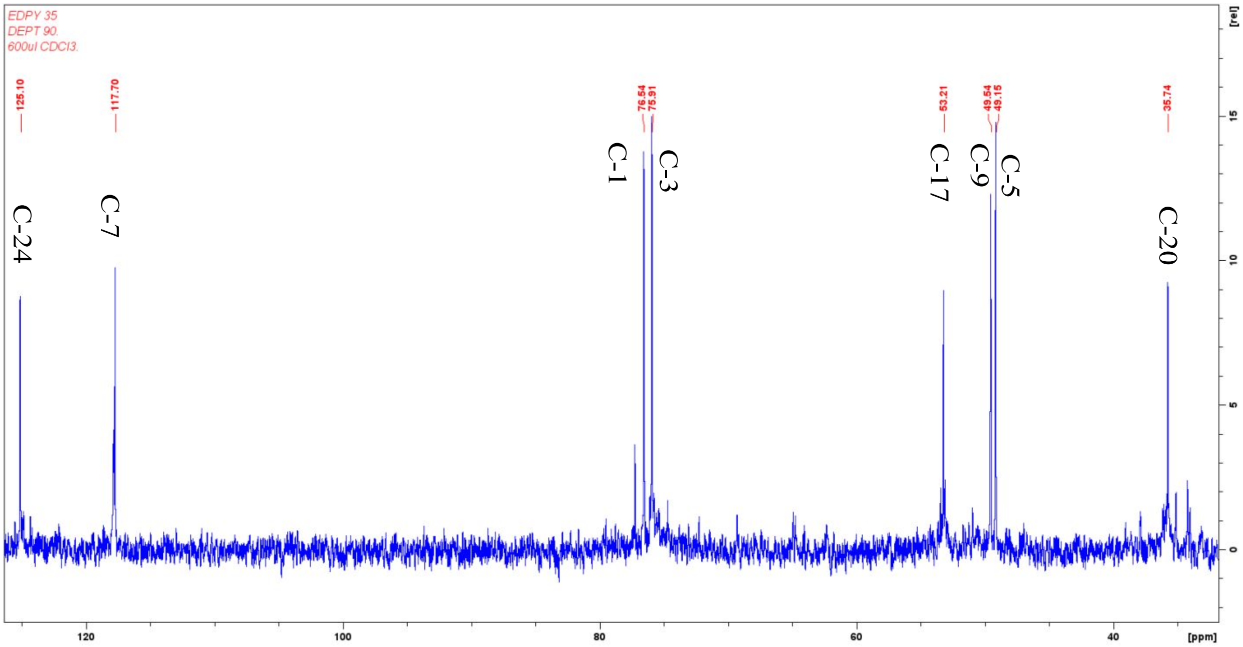

c)

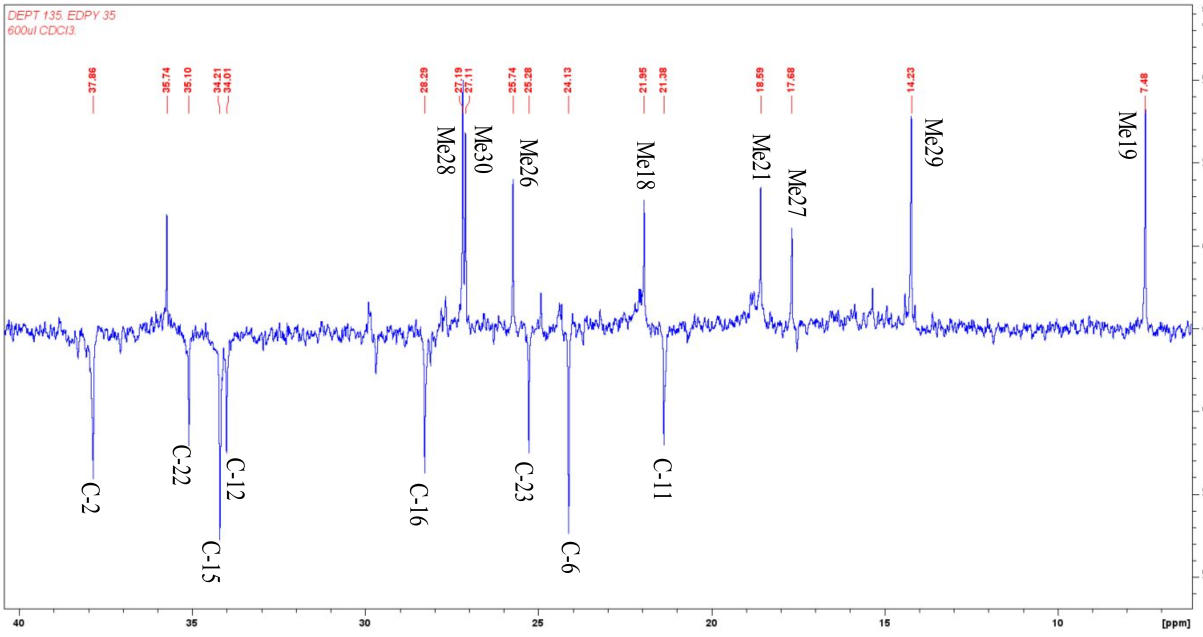

Figure S6. Selected J-resolved spectra of oddurensinoid **B**. a) Plot of the H-3 and H-3 regions shows the resolved H-3 and H-1 strips that are well resolved; b) plot of the up-field regions that help to resolve the a few

important methyl groups; c) plot of extract of H-2_α from J-resolved spectrum; d) plot of extract of H-2_β from J-resolved spectrum.

(a)

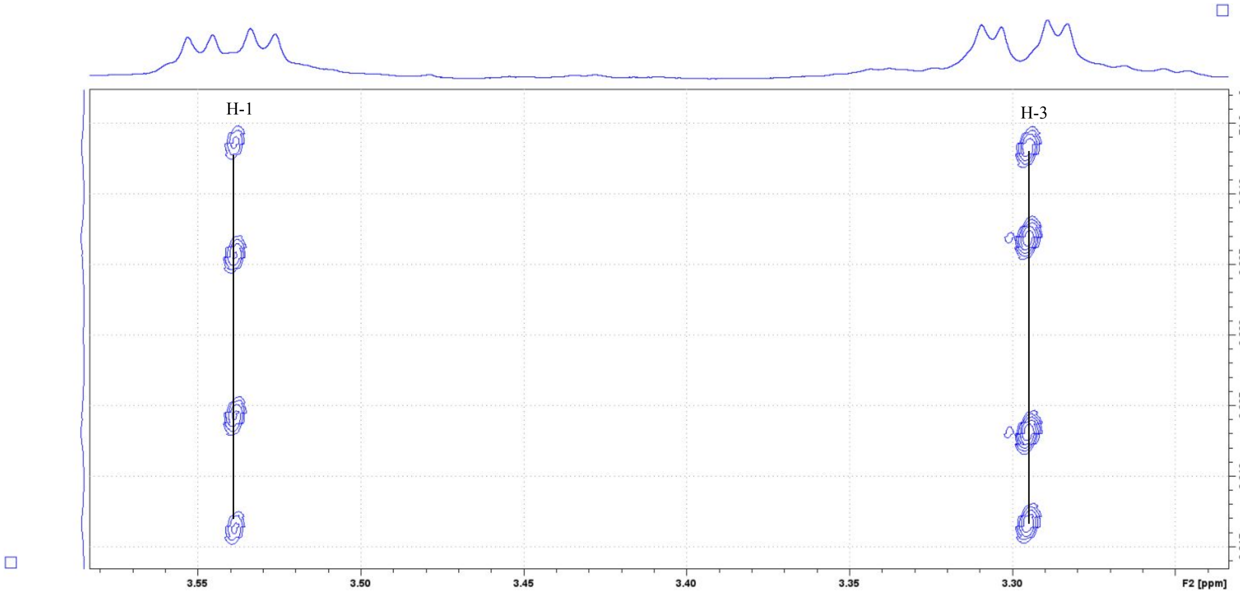

(b)

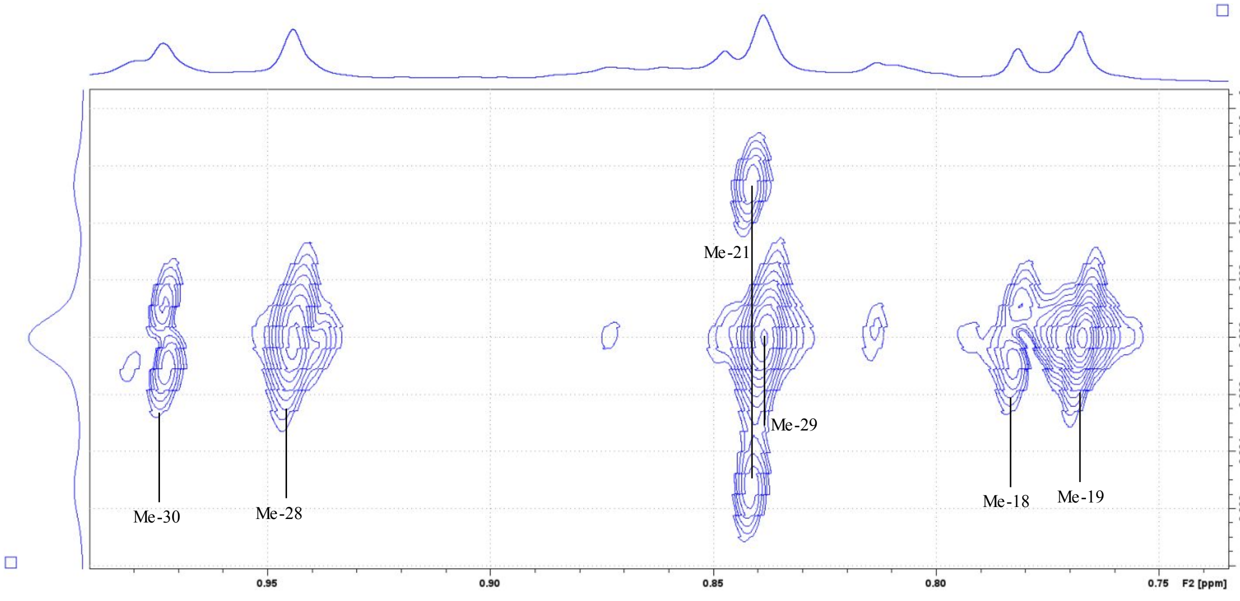

(c)

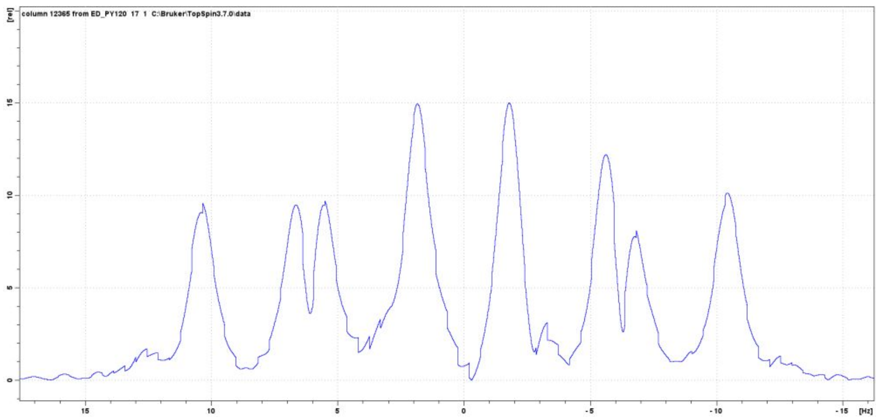
column 12365 from ED_PY120 17 1 C:\Bruker\TopSpin3.7.0\data
[rel]
[Hz]

(d)

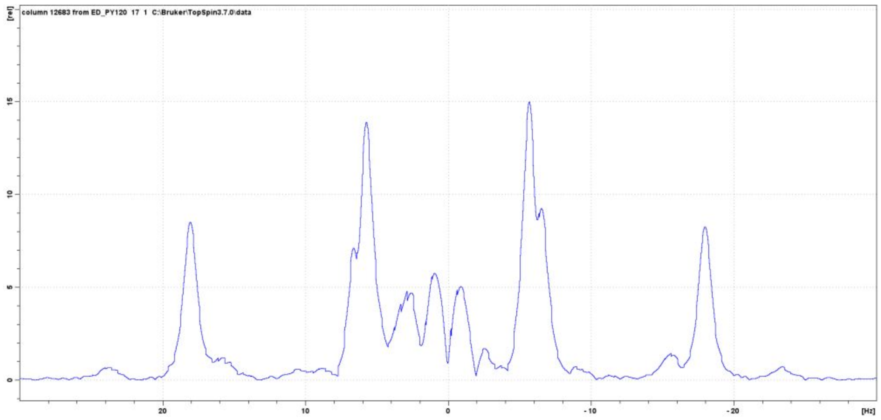
column 12683 from ED_PY120 17 1 C:\Bruker\TopSpin3.7.0\data
[rel]
[Hz]

Figure S7. Plot of selected regions of ^{13}C NMR spectra of for oddurensinoid **K** with the assignments labelled.

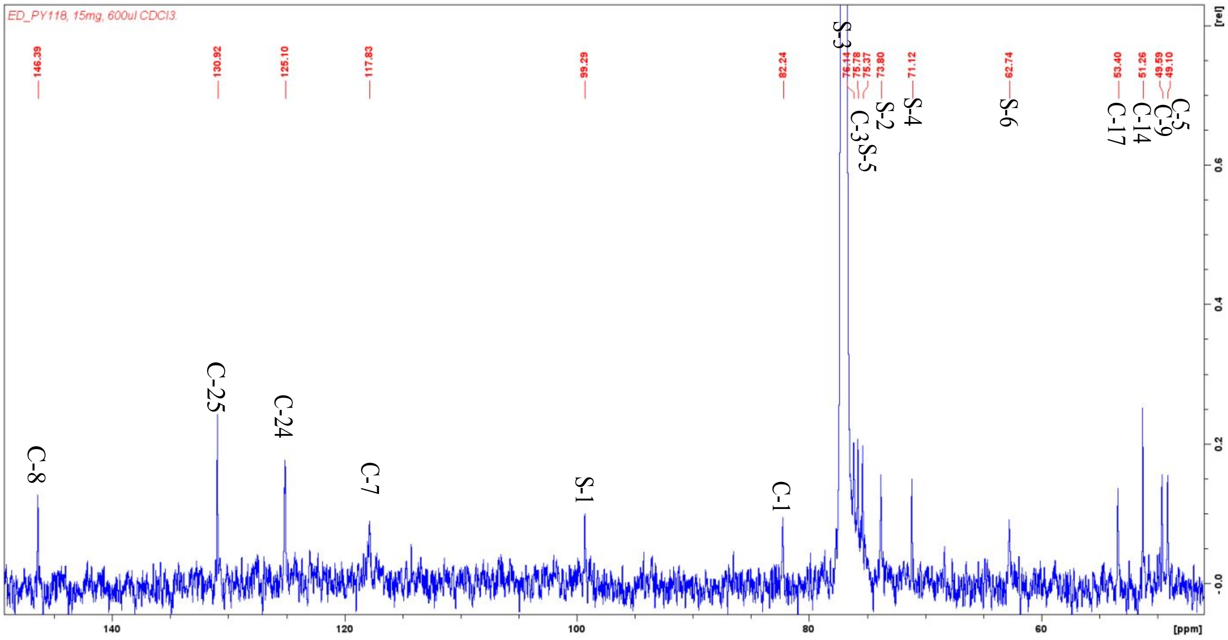

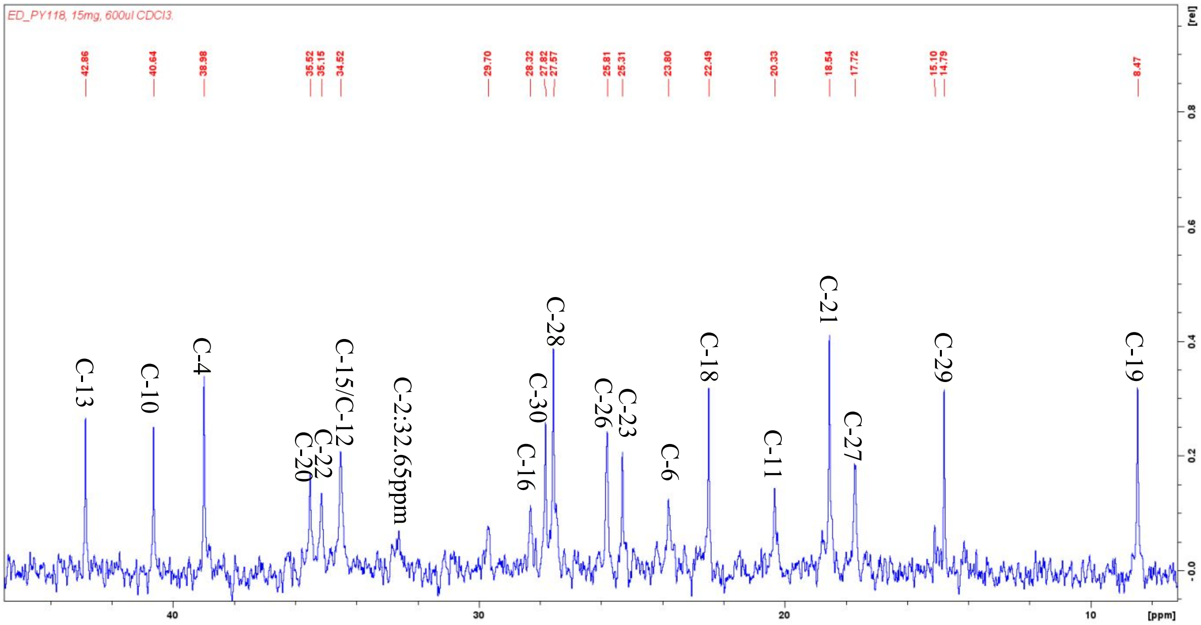

Figure S8. Plots of selected regions of COSY (a) and NOESY (b) spectra for oddurensinoid **K** illustrating the assignments.

(a) COSY:

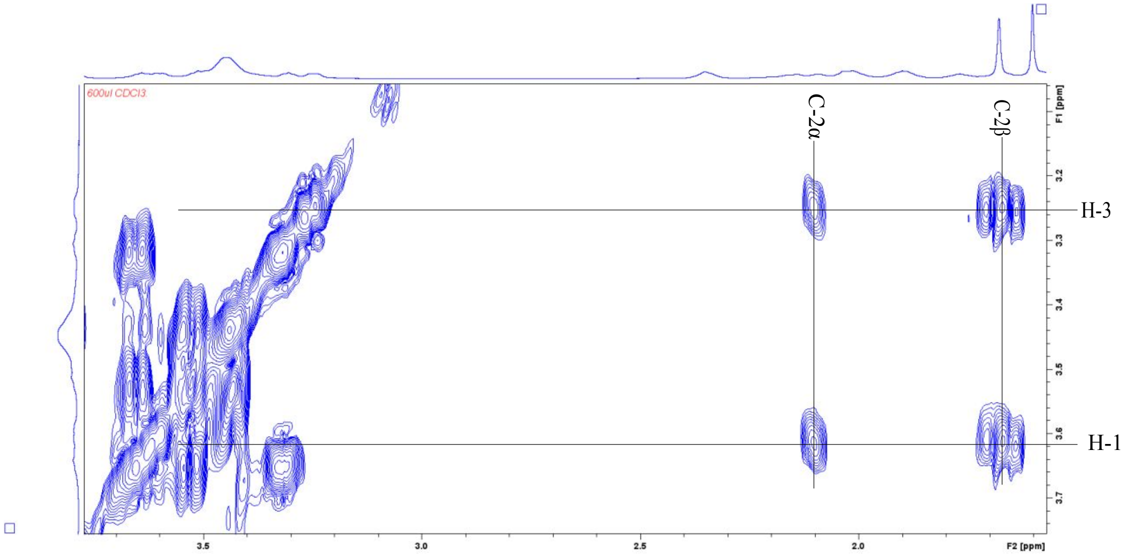

(b) NOESY:

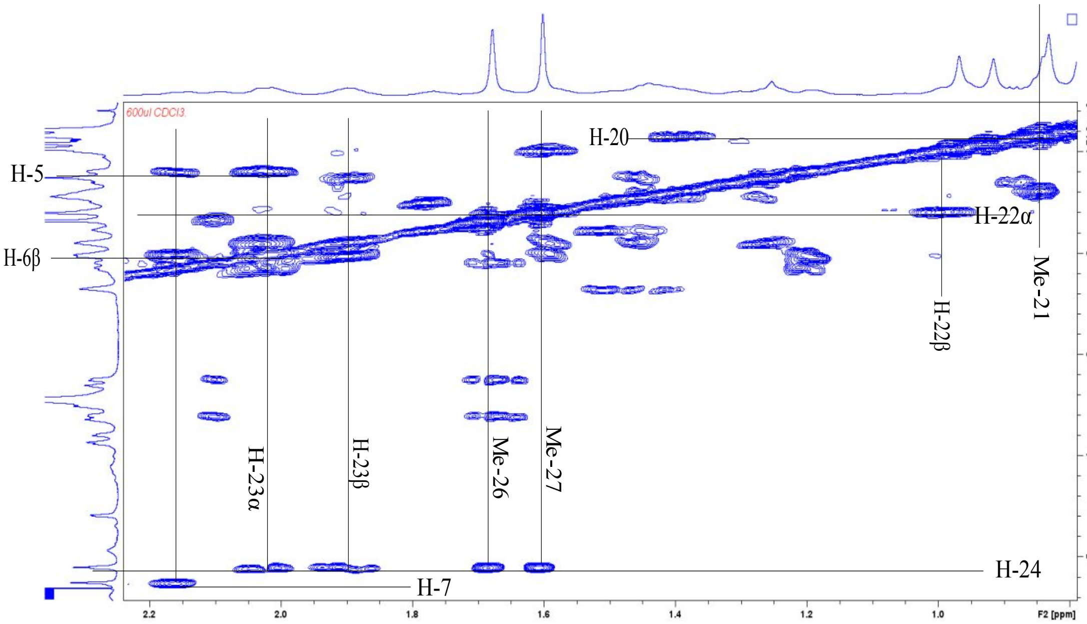

Figure S9. Plots of selected regions of HMBC spectra for oddurensinoid **K** elucidating the assignment of hexose connectivity.

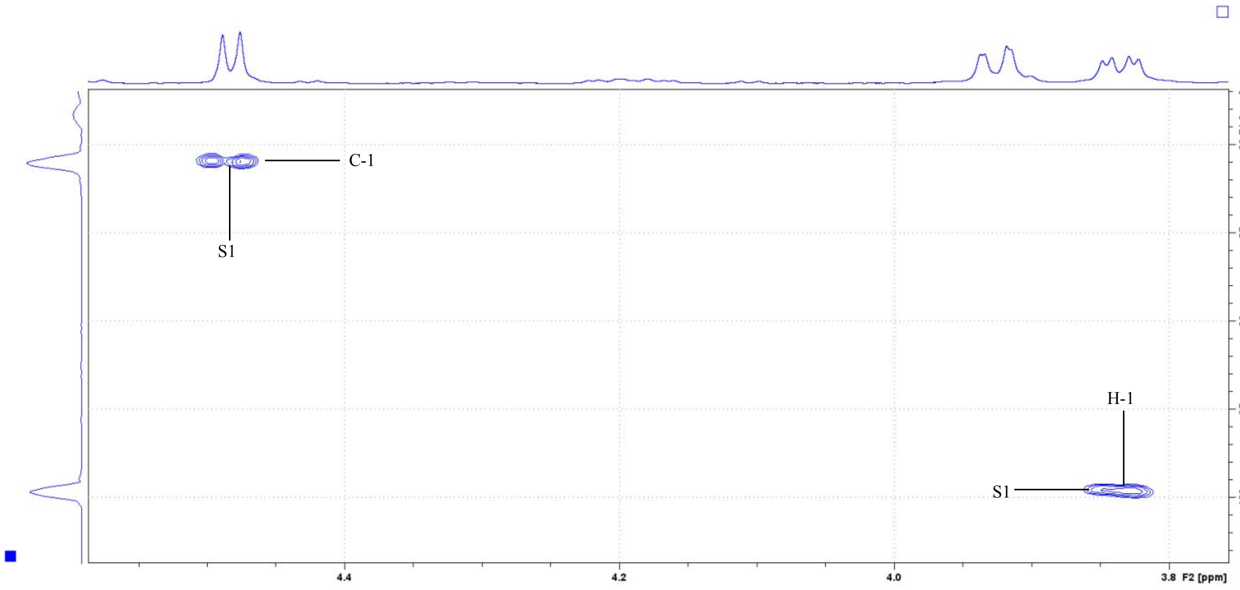

Figure S10. Full 1D ^1H (600 MHz) NMR spectra of for oddurensinoid **B**.

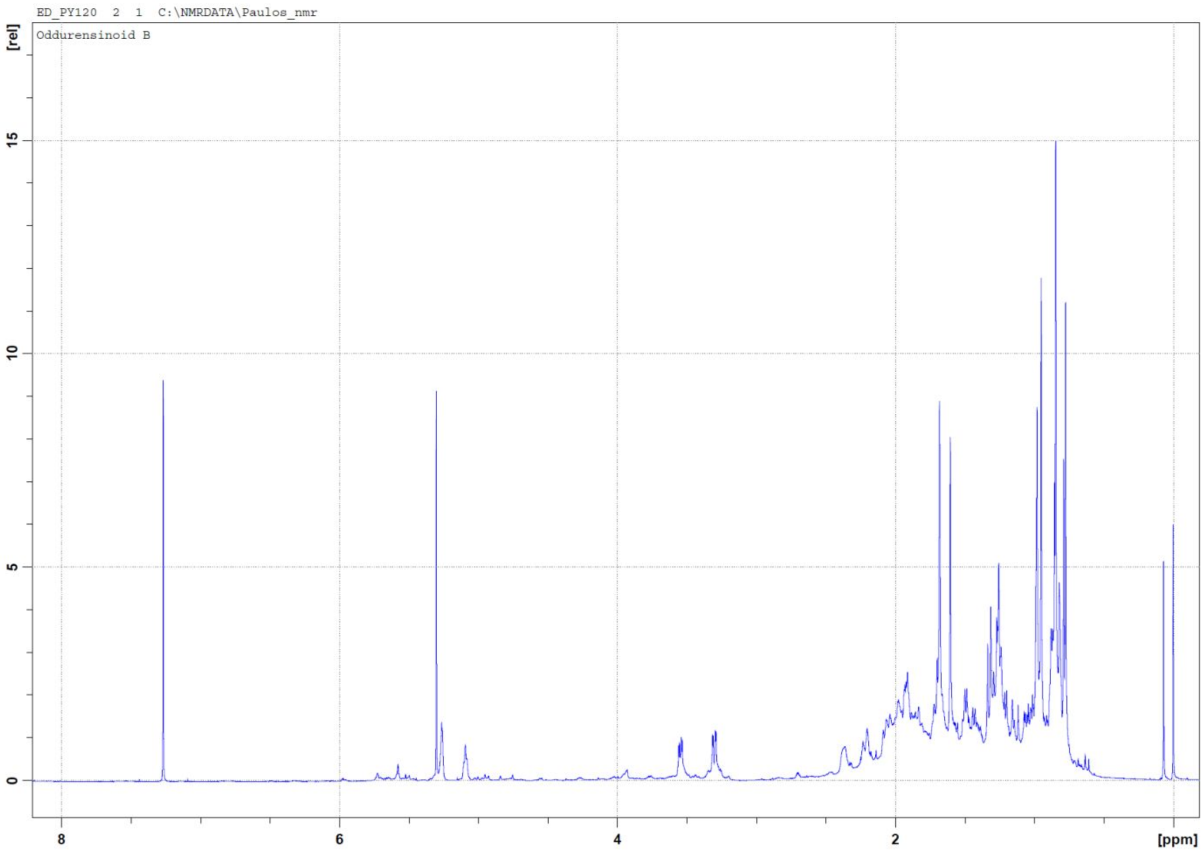

Figure S11. Full 1D ^{13}C NMR (150 MHz) spectra of for oddurensinoid **B**.

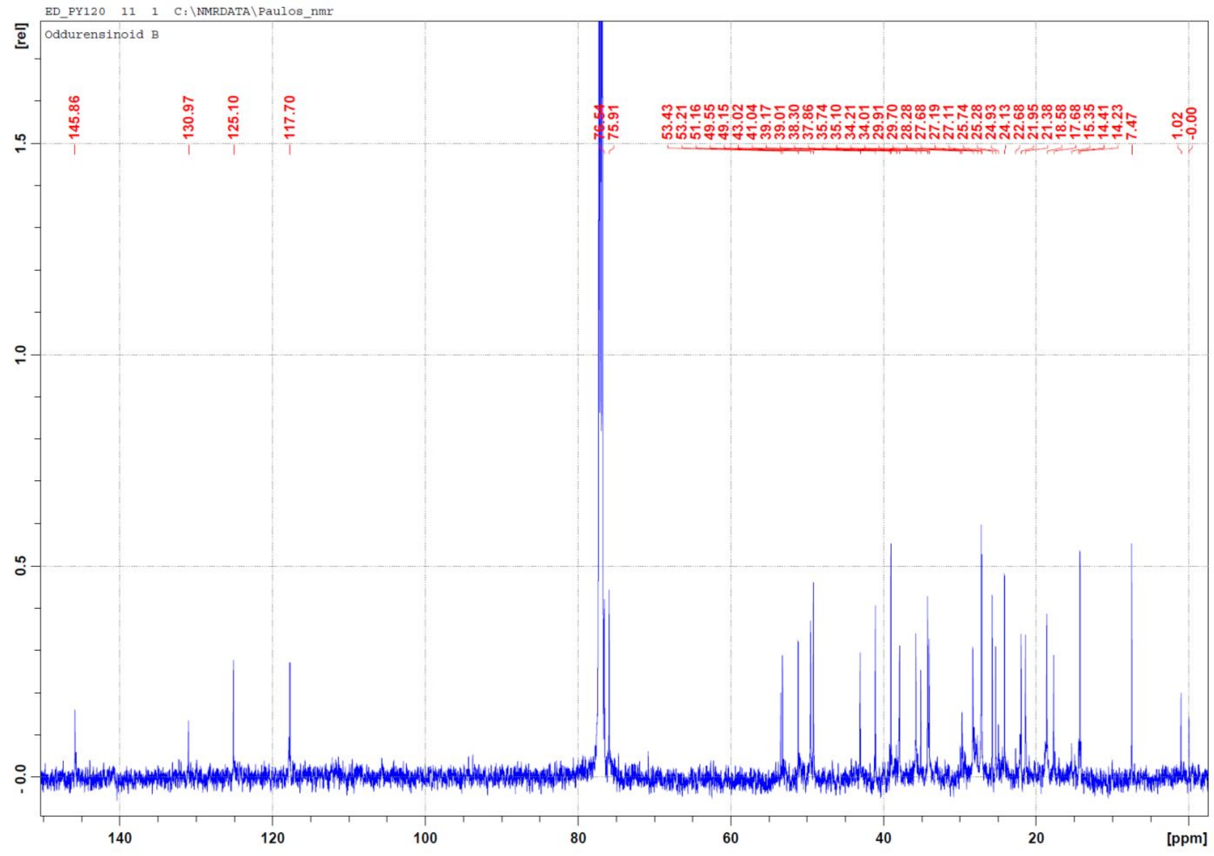

Figure S12. Full 1D ^{1}H (600 MHz) NMR spectra of for oddurensinoid **H**.

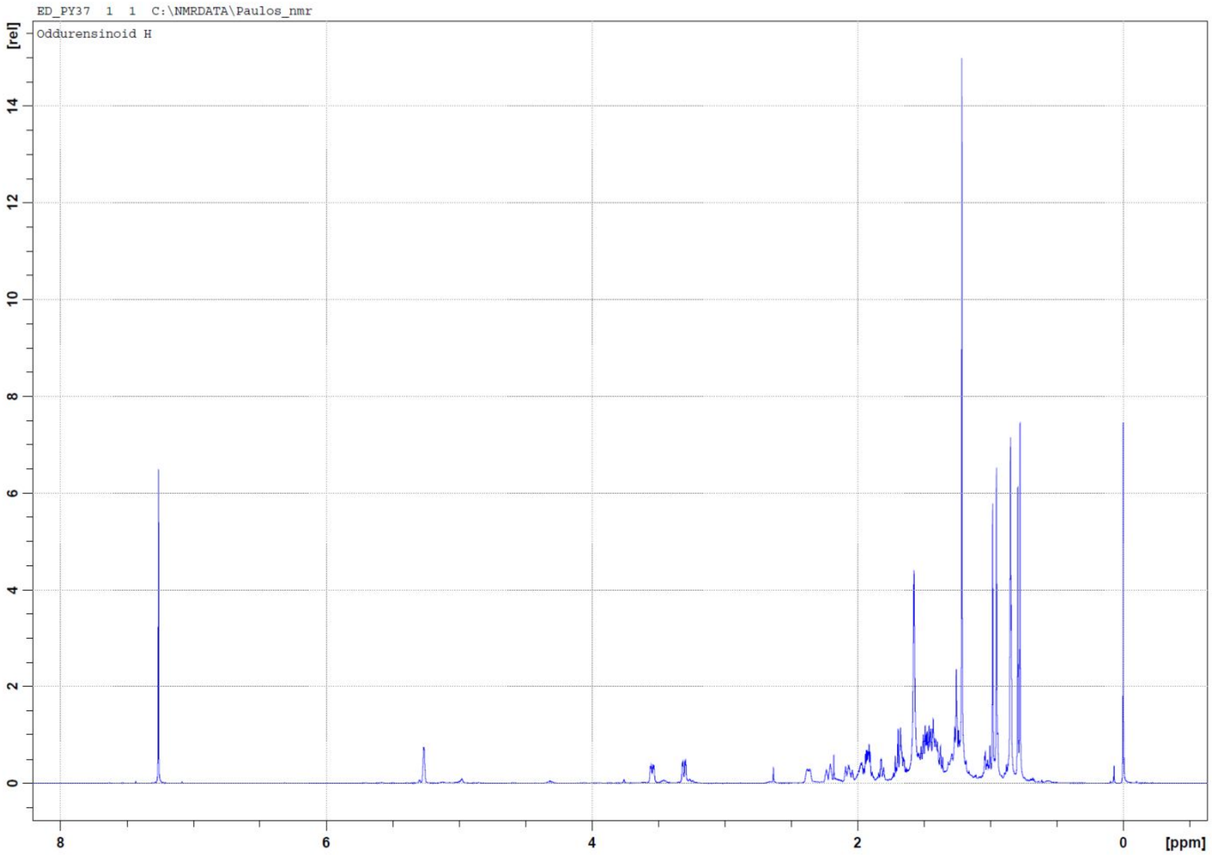

Figure S13. Full 1D ^{13}C NMR (150 MHz) spectra of for oddurensinoid **H**.

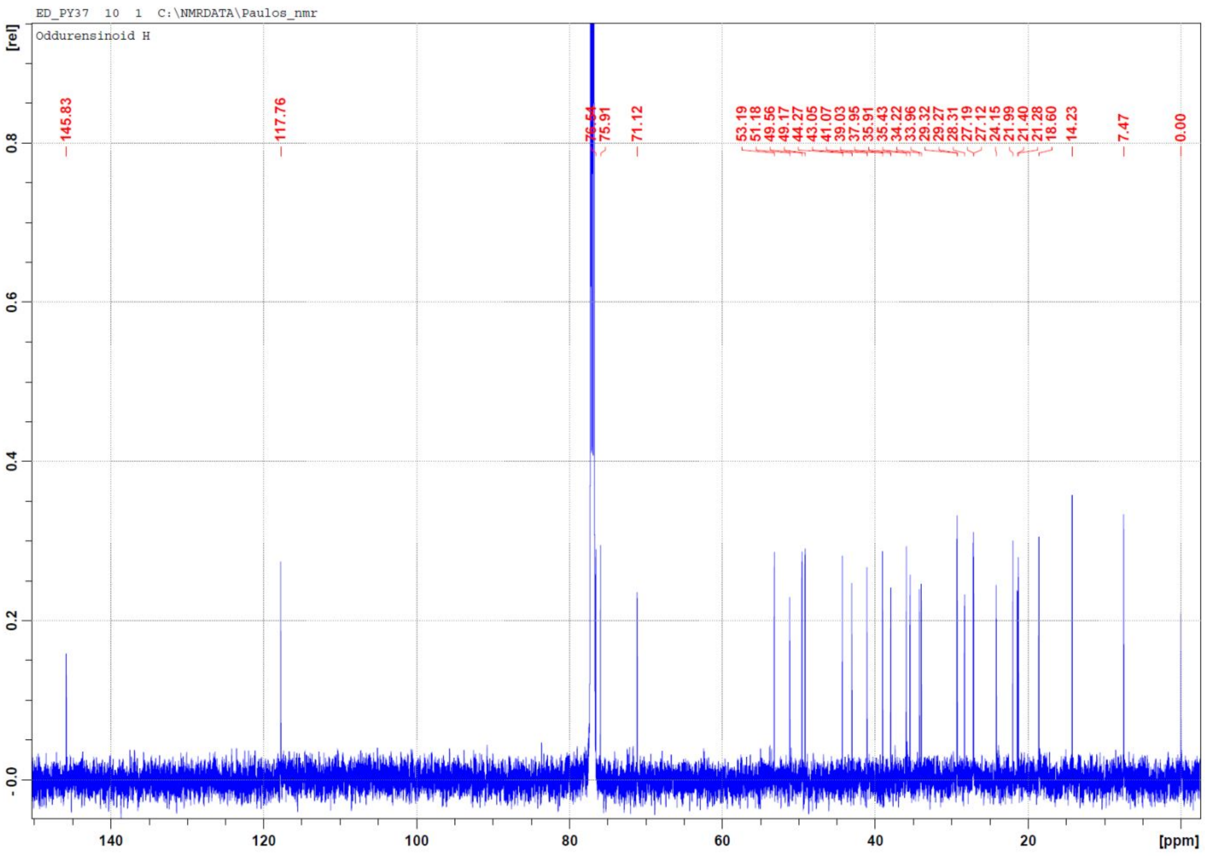

Figure S14. Full 1D ^{1}H (600 MHz) NMR spectra of for oddurensinoid **K**.

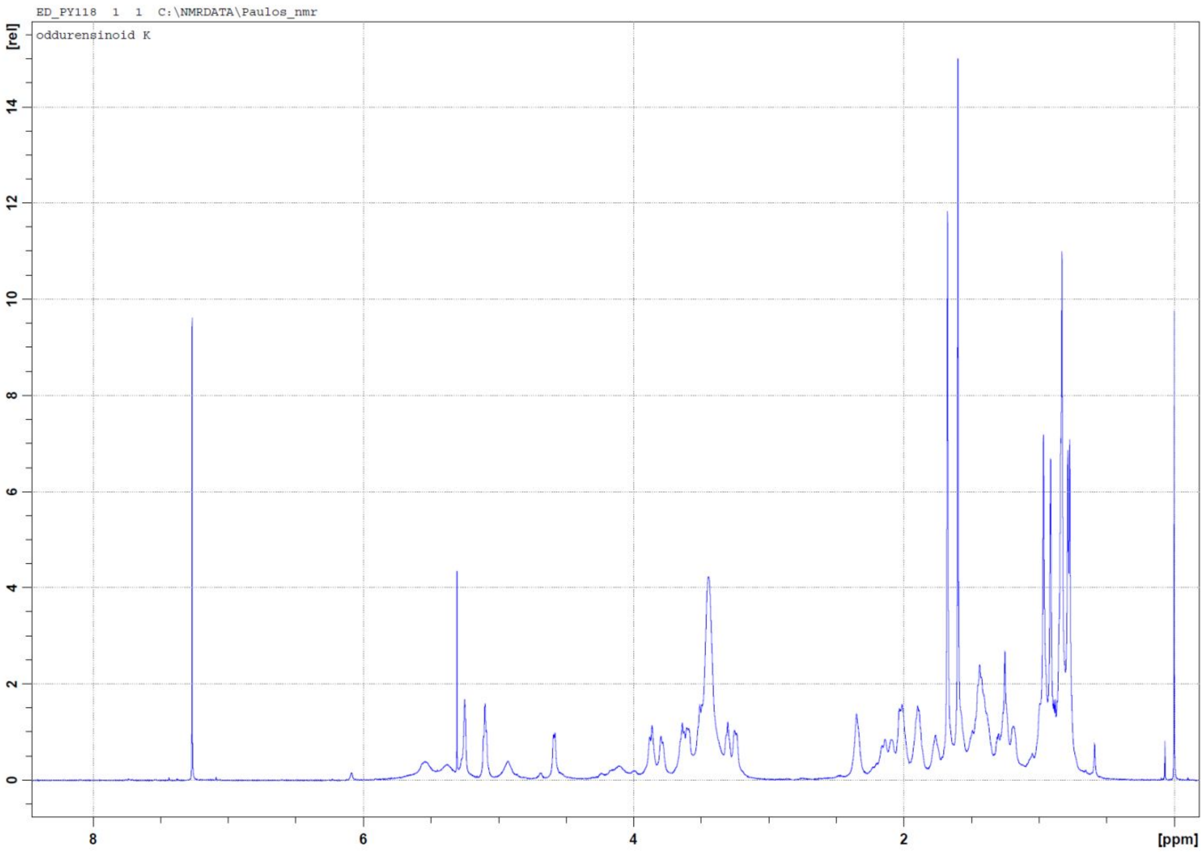

Figure S15. Full 1D ^{13}C NMR (150 MHz) spectra of for oddurensinoid **K**.

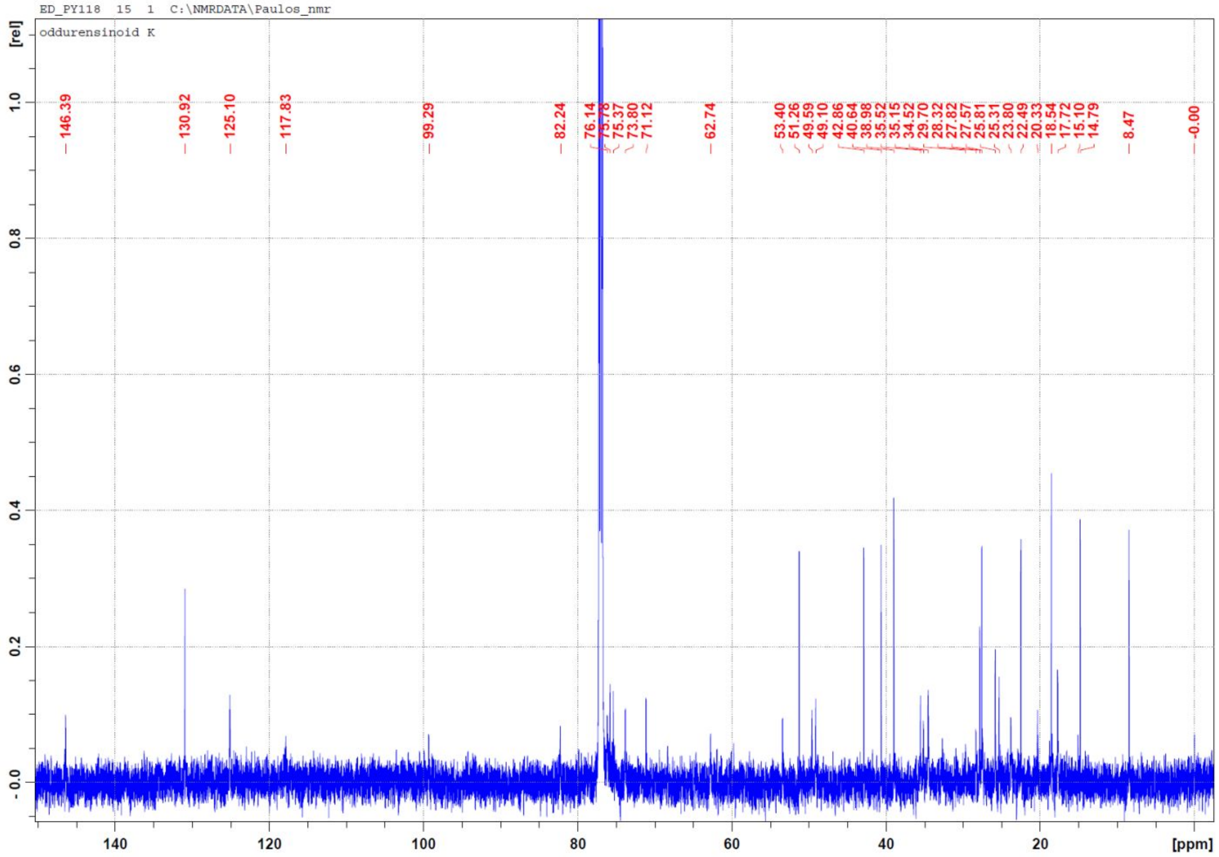

Supplement: Supplementary file 1 [file ao5c03203_si_001.pdf]
